# Striatal resting-state connectivity after long-term diacetylmorphine treatment in opioid-dependent patients

**DOI:** 10.1093/braincomms/fcac275

**Published:** 2022-10-26

**Authors:** Anna-Chiara Schaub, Marc Vogel, Sophie Baumgartner, Undine E Lang, Stefan Borgwardt, André Schmidt, Marc Walter

**Affiliations:** Department of Psychiatry (UPK), University of Basel, 4002 Basel, Switzerland; Department of Psychiatry (UPK), University of Basel, 4002 Basel, Switzerland; Department of Psychiatry (UPK), University of Basel, 4002 Basel, Switzerland; Department of Psychiatry (UPK), University of Basel, 4002 Basel, Switzerland; Department of Psychiatry and Psychotherapy, University of Lübeck, 23562 Lübeck, Germany; Department of Psychiatry (UPK), University of Basel, 4002 Basel, Switzerland; Department of Psychiatry (UPK), University of Basel, 4002 Basel, Switzerland; Psychiatrische Dienste Aargau, Windisch, Switzerland

**Keywords:** diacetylmorphine, heroin maintenance, long-term effects, resting-state functional MRI, striatum

## Abstract

New treatment approaches for opioid-dependent patients include injectable opioid agonist treatment with diacetylmorphine. While evidence has shown beneficial clinical effects of diacetylmorphine, it is still not clear how long-term diacetylmorphine treatment affects the brain and whether functional brain changes are accompanied by clinical improvements. Therefore, this prospective case-control study focuses on long-term effects of diacetylmorphine on resting-state functional connectivity.

We included opioid-dependent patients (*N* = 22, age range 33–58, 16 males) treated with diacetylmorphine and healthy controls (*N* = 9, age range 27–55, 5 males) that underwent two MRI assessments approximately nine years apart. For the patients, the assessments took part shortly after the diacetylmorphine intake to be able to explore changes in resting-state functional connectivity in brain regions related to the stage of binge and intoxication (caudate, putamen, nucleus accumbens).

A cluster in the right superior frontal gyrus was detected, showing over nine years an increase in functional connectivity originating from the left caudate and the left accumbens in patients but not in healthy controls. These connectivity changes in patients were related to the duration of the diacetylmorphine treatment at the follow-up, indicating smaller increases in functional connectivity with longer treatment duration (*r* = 0.63, *P* < 0.01).

These results suggest that long-term diacetylmorphine treatment in opioid-dependent patients increases fronto-striatal connections, an effect that is linked to the duration of the treatment duration. Future research needs to further address the wide-ranging effects of diacetylmorphine on brain functioning and deepen the understanding of their clinical relevance.

## Teaser

Schaub et al. report functional brain changes in patients receiving long-term diacetylmorphine treatment. Over nine years, fronto-striatal resting-state functional connectivity increased in patients compared to healthy controls. This effect was partly related to the treatment duration, indicating smaller increases in with longer treatment.

## Graphical Abstract

**Graphical abstract:**
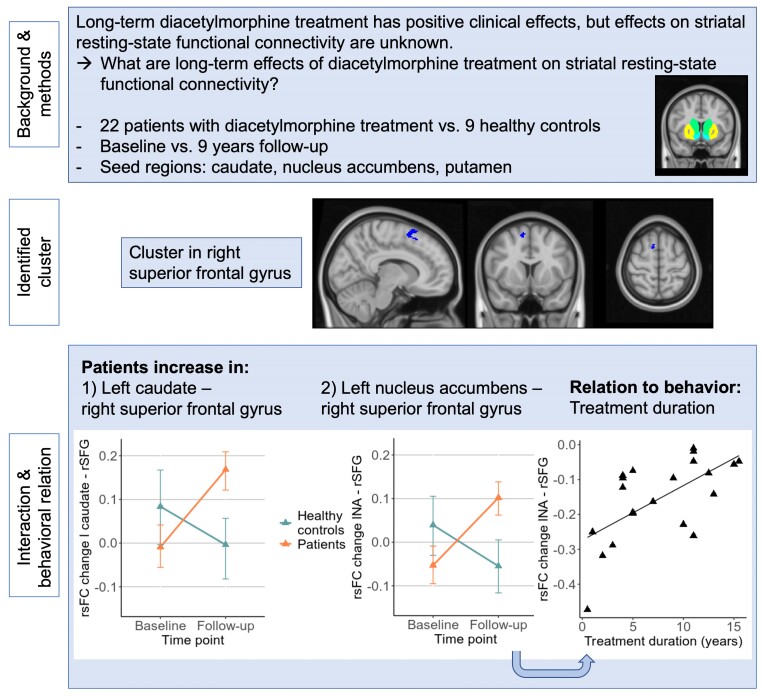

## Introduction

The ongoing worldwide opioid crisis is of great importance as the direct burden of disability-adjusted life years is highest for opioid-dependent subjects in comparison to other illicit drugs.^1^ For this crisis, by its changing nature, it is challenging to find effective and adequate responses such as prevention and treatment approaches.^2^ To reduce the burden of opioid use disorder (OUD), various treatments exist to date including different options of opioid-agonist treatments (OAT). As not all patients respond to these first-line treatments, diacetylmorphine (DAM) as additional treatment approach for treatment-refractory chronic heroin-dependent patients was introduced.^3^ DAM is a cost-effective alternative for non-responding patients to other OAT such as methadone or buprenorphine.^4^ DAM has beneficial clinical effects by increasing treatment retention and reducing illicit opioid use, but necessitates close monitoring to avoid overdoses and seizures.^5–7^ Studies found additional beneficial effects such as improved physical and mental health,^8, 9^ improved employment status^6^ and reduced criminal activity even though it is not clear if this specific effect is stronger than in oral methadone.^10^

Regarding the underlying neural mechanisms of OUD, research sheds light on the importance of the reward system early in the development of the disease.^11^ The reward system is crucially mediating the binge or intoxication stage shortly after drug intake and is as such postulated to be predominant at early stages of drug addiction.^11, 12^ The binge/intoxication stage involves dopaminergic pathways in the basal ganglia, whereby the ventral striatum (nucleus accumbens) plays a key role in the acute hedonic effects and the dorsal striatum (putamen and nucleus caudate) in subsequent habit formation,^13^ contributing both to compulsive substance seeking.^14^ Later in the course of drug development, stages of withdrawal along with negative affect and as a third stage craving or preoccupation, which imply regions such as the amygdala and prefrontal and orbitofrontal regions, respectively, motivate continued drug intake.^11^ As a circular cascade, these later stages of withdrawal and craving in turn initiate new rounds of drug consumption through aberrant fronto-striatal interactions.^15, 16^

OUD has been associated with structural and functional alterations in the striatum. Decreased volume of the nucleus accumbens and putamen was found in OUD patients compared to healthy controls.^17, 18^ Notably, volumes in nucleus accumbens and putamen were negatively related to depression severity^17^ and the duration of heroin abuse.^18^ However, no volumetric differences in the striatum have been reported in another study with heroin-dependent patients.^19^ A resting-state functional MRI study further showed decreased amplitude of low-frequency fluctuation (ALFF) in OUD patients in the right caudate, anterior cingulate cortex and superior frontal cortex, whereas ALFF values in the right caudate were negatively associated with the duration of heroin use and daily heroin dose.^20^ Furthermore, compared with non-relapsers, heroin relapsers showed increased regional homogeneity in the right caudate that was positively related to heroin relapse rates and craving responses.^21^ OUD is also associated with weaker fronto-striatal resting-state functional connectivity^22^ which has also been shown in other substance use disorders.^23, 24^ In nicotine-dependent subjects, smoking and its subsequent reduction in craving are linked to increases in fronto-striatal rsFC and more precisely in the dorsolateral prefrontal cortex.^25^ Interestingly, the opioid receptor antagonist naltrexone leads to lower opioid wanting by increasing fronto-striatal connectivity also affecting the dorsolateral prefrontal cortex.^26^ We have previously shown that acute DAM administration still increased dorsal striatal connectivity in OUD patients, which correlated positively with the plasma level of morphine and the subjective feeling of rush.^27^ These results show that even after prolonged heroin intake, the drug still releases rewarding effects reflected by increased striatal activity, although previous research had questioned the prolonged involvement of dopamine located especially in the striatum in addiction.^28^ Not much evidence is available regarding long-term DAM treatment and its effects on the brain. A voxel-based morphometry analysis showed increased volume of the right caudate and reductions in the right amygdala, anterior cingulate cortex and the orbitofrontal cortex and was able to link these effects to drug-related measures such as the DAM dose.^29^ However, the interpretation of the results must be done with caution since no control sample with healthy subjects was included.

Long-term effects of DAM are important to investigate since patients often remain in treatment for a protracted period and termination rates are low.^5, 30^ Beside its beneficial clinical effects, it is of great interest to understand how DAM treatment affects the brain and especially brain functioning. This study therefore focuses on effects of DAM on rsFC. We conducted a resting-state functional MRI assessment shortly after the DAM intake and compared changes over a nine-year period to changes in healthy control subjects. Based on previous research, we expected rsFC increases in OUD patients compared to healthy controls in connections originating from the striatum to the prefrontal cortex. Furthermore, we explored whether functional brain changes are linked to clinical and behavioural measures expecting a negative association with craving as its association with fronto-striatal connections have previously been shown^31^ and furthermore an association with the duration of the opioid use and DAM dose.

## Materials and methods

This study represents a nine-year follow-up analysis of a randomized placebo-controlled, crossover trial exploring acute DAM effects in OUD patients.^27, 32, 33^ First follow-up results on the long-term effects of DAM on brain volume changes in OUD patients are reported elsewhere.^29^ Here we report findings of long-term DAM effects in OUD patients compared to general aging effects in healthy controls on striatal rsFC.

### Participants

Out of 27 patients with OUD that were included in the original baseline study,^32^ 22 patients completed a nine-year follow-up MRI assessment. For initial inclusion, patients had to be older than 18 years and had a history of OUD with current injectable opioid treatment (> 6 months). The dose had to be unchanged during the previous three months. Patients were excluded when they had a positive alcohol breathalyzer test and additional physical or psychiatric diseases including severe substance use disorders. Tobacco use and alcohol or drug abuse such as cocaine and cannabis led not to exclusion. Patients had at least two unsuccessful treatments for OUD and all patients were enrolled in a standardized OAT program (JANUS, University of Basel, Department of Psychiatry [Universitäre Psychiatrische Kliniken], Switzerland), which includes the prescription of DAM and psychosocial treatment. History of heroin and other illicit substance use was assessed, and behavioural measures related to heroin dependence such as duration of dependence at baseline (in years), age of first use, duration of the DAM treatment at baseline (in years) and the daily opioid dose (baseline and follow-up in mg) were recorded.

Beside OUD patients, nine out of 20 healthy controls from an initial baseline sample were included in this nine-year follow-up MRI study. Healthy controls had no psychiatric or neurological diagnoses and no family history of psychiatric illness.

The study was approved by the local ethics committee (Ethikkommission Nordwest und Zentralschweiz) according to the Declaration of Helsinki, and all participants gave written informed consent before inclusion in the study.

### Study design

Patients administered DAM and underwent an MRI session 20 minutes after the intake. Depressive symptoms and craving were assessed shortly after the DAM intake using the Beck depression inventory (BDI-II)^34^ and the heroin craving questionnaire (HCQ),^35^ respectively. Medication and side consummation was tracked during the study interval. At the nine years follow-up assessment, the same MRI session, the BDI-II and HCQ were assessed again. Healthy controls did not receive any treatment but underwent two MRI sessions at a nine-year interval.

### MRI assessment

Patients and healthy controls underwent an MRI session including structural and resting-state functional MRI sequences at the baseline and follow-up assessment using a 3T MAGNETOM VERIO scanner (SIEMENS, Erlangen, Germany) with a 12-channel radiofrequency head coil. Foam pads across the forehead were used to minimize head movement. For the 5 min resting-state sequence, subjects were instructed to lie in the scanner with eyes open, to think of nothing in particular, and not to fall asleep. We used a gradient echo planar imaging (EPI) sequence (TR = 2000 ms, TE = 28 ms, slice thickness 3.3 mm, field of view 228 mm, flip angle 82°, 152 volumes, voxel size 3.6 × 3.6 × 3.3 mm, bandwidth of 2694 Hz/pixel) and for anatomical reference, a 3D whole-brain T1-weighted magnetization prepared rapid acquisition gradient (MPRAGE) sequence was applied (176 slices, field of view 256 mm, TR = 2000ms, TE = 3.37 ms, flip angle 8°, 1 mm slice thickness, 1 × 1 × 1 mm voxel size, bandwidth of 200 Hz/pixel).

### Resting-state analysis

Functional MRI data were processed and analysed using the CONN toolbox (19.c, http://www.nitrc.org/projects/conn),^36^ an open-source Matlab/SPM-based software. For preprocessing, a default pipeline for volume-based analysis was applied including realignment and unwarping (subject motion estimation and correction), slice timing correction, outlier detection using ART-based identification, segmentation into grey and white matter and CSF, direct normalization into standard Montreal Neurological Institute (MNI) space and smoothing (gaussian kernel FWHM = 6 mm) in the presented order. Denoising was done using linear regression of potential confounding effects implemented with an anatomical component-based noise correction procedure (aCompCor). It includes subject motion parameters, scrubbing,^37^ noise components from white matter and cerebrospinal areas and session effects. Finally, linear detrending and temporal band-pass filtering (0.0008 < *f* < 0.09 Hz) were applied.

### Statistical analysis

**Figure 1 Seed regions of seed-to-voxel analysis for binge/intoxication stage.**
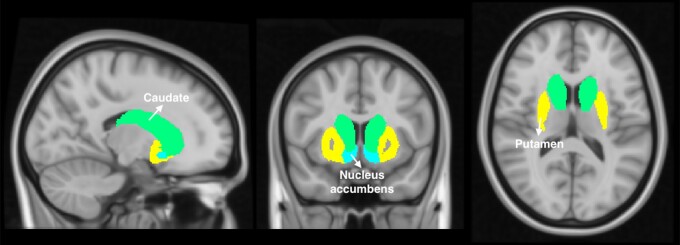
Binge regions consisted of the bilateral caudate, putamen and nucleus accumbens according to the Harvard-Oxford subcortical structural atlas as implemented in conn.

Brain regions that have been linked to the binge/intoxication stage of dependence were selected as seeds such as the dorsal striatum (bilateral caudate, putamen) and the nucleus accumbens (Fig. 1).^11^ Brain maps of bivariate correlation coefficients (fisher transformed) were calculated for each subject, time point, and seed. The general linear model (GLM) was set to an 2 × 2 mixed ANCOVA interaction comparing OUD patients and healthy controls over time from baseline to the follow-up assessment. All six seeds of the binge stage were jointly included as sources to avoid problems of multiple testing; targets were voxels covering the whole brain. Change scores of smoking (quantity at baseline—follow-up) and a score of cocaine, benzodiazepine and cannabis use (−1: baseline no/follow-up yes, 0: no change, 1: baseline yes/fallow-up no) were demeaned across all subjects and included as covariates to avoid confounding effects. To define clusters of interest, an uncorrected voxel threshold of *P* < 0.001 was applied and results were considered significant with an FDR-corrected *P* < 0.05 cluster-level threshold. To further validate our findings, we conducted an additional analysis by splitting the sample of patients into two separate samples (half 1: 1 0 1 0 1 0, etc., half 2: 0 1 0 1 0 1, etc.) and run two separate analyses comparing both subgroups with the healthy controls including the same covariates (2 × 2 mixed ANCOVA).

### Behavioral and exploratory analysis

Comparisons between study groups in behavioral and clinical measures were conducted using chi-squared tests, two sample *t*-tests and non-parametric Wilcoxon signed rank tests.

Exploratory analyses were added to investigate if behavioural (BDI-II, HCQ) and heroin-related measures (duration of DAM treatment, duration of heroin dependence, age of first use, daily opioid dose at baseline and follow-up) were related to significant rsFC changes (baseline—follow-up). Therefore, linear models were set including different covariates (age and gender) and an analysis of variance (ANOVA, type III) was run over the model with the best fit. In addition, spearman correlations were conducted in the heroin group including change scores of behavioural measures as mentioned above and changes in rsFC. As this analysis is of exploratory nature, no corrections for multiple testing were applied. Possible bivariate outliers were detected using mahalanobis’ distance (MD) and participants were removed from correlations when above the cutoff (chi-square distance with *P* = 0.95, df = 2). All statistical analyses were carried out using R. Effect sizes were based on cohens’ D or Cliffs delta for ordinal data.

## Results

### Participants

OUD patients and healthy controls did not differ significantly in age and gender, however, differences in clinical and behavioural measures occurred such as in education, smoking, cocaine use and depressive symptoms (Table 1). In OUD patients, the DAM dose and delivery form changed in some patients but not all; some patients switched from intravenous application to oral and two patients did no longer receive any DAM at the follow-up. The mean intravenous dose dropped significantly at the follow-up (*t*(21) = 2.47, *P* < 0.05, *d* = 0.7, 95%-confidence interval (CI) −0.2–1.57), but when combining intravenous and oral doses at follow-up, the dose was not significantly different to the initial dose at baseline (*t*(21)=−0.11, *P* = 0.91, *d* = 0.04, 95-CI: −0.8− 0.87). Depressive symptoms in the patients decreased over the study period (*V* = 163.5, *P* < 0.5, delta = 0.28, 96%-CI: −0.09–0.58), while craving (HCQ) did not change significantly (*V* = 139.5, *P* = 0.41, delta = 0.06, 96%-CI: −0.29–0.4). Depressive symptoms in healthy controls did not change over time (*V* = 9, *P* = 0.44, delta = −0.04, 96%-CI: −0.51–0.45).

**Table 1:** Sociodemographic and behavioral characteristics of the study sample

	Patients (*N* = 22)	Controls (*N* = 9)	Comparison
**Sociodemographic measures**			
Age baseline, mean (SD)	42.32 (5.41)	39.33 (9.42)	*T*(10.23) = 0.89, *P* = 0.39
Age follow-up, mean (SD)	50.95 (5.44)	48.22 (9.11)	*T*(10.42) = 0.84, *P* = 0.42
Gender, male/female	16/6	5/4	χ^2^ = 0.26, *P* = 0.61
Handedness, right/left	22/0	9/0	-
Education, mean (SD)	10 (1.14)	13.44 (4.1)	*T*(8.62)=−2.48, ***P* = 0.04**
Smoking baseline, mean per day (SD)	22.23 (9.72)	12.22 (7.01)	*T*(20.65) = 3.20, ***P* < 0.01**
Smoking follow-up, mean per day (SD)	17.32 (8.32)	5.33 (7.4)	*T*(16.72) = 3.94, ***P* < 0.01**
Smoking, mean change (BL-FU) (SD)	4.91 (7.94)	6.89 (11.44)	*T*(11.3)=−0.47, *P* = 0.64
Benzodiazepine use baseline, yes/no	6/16	0/9	χ^2^ = 1.55, *P* = 0.21
Benzodiazepine use follow-up, yes/no	7/15	1/8	χ^2^ = 0.55, *P* = 0.46
Benzodiazepine use change (BL-FU)^a^	4/15/3	1/8/0	χ^2^ = 1.79, *P* = 0.41
Cocaine use baseline, yes/no	9/13	0/9	χ^2^ = 3.39, *P* = 0.07
Cocaine use follow-up, yes/no	10/12	0/9	χ^2^ = 4.14, ***P* = 0.04**
Cocaine use change (BL-FU)^a^	4/15/3	0/9/0	χ^2^ = 3.7, *P* = 0.16
Cannabis use baseline, yes/no	8/14	2/7	χ^2^ = 0.12, *P* = 0.73
Cannabis use follow-up, yes/no	8/14	1/8	χ^2^ = 0.94, *P* = 0.33
Cannabis use change (BL-FU)^a^	4/14/4	1/6/2	χ^2^ = 0.26, *P* = 0.88
**Heroin-related measures**			
Age of first heroin use (years), mean (SD)	19.27 (3.4)	N.A.	N.A.
Duration heroin use baseline (years), mean (SD)	22.05 (5.55)	N.A.	N.A.
Duration DAM baseline (years), mean (SD)^b^	7.59 (4.64)	N.A.	N.A.
Daily heroin dose at BL, iv (mg), mean (SD)	345.91 (129.23)	N.A.	N.A.
Daily heroin dose at FU, iv (mg), mean (SD)	234.55 (182.44)	N.A.	N.A.
Daily heroin dose at FU, total (mg), mean (SD)^c^	351.59 (167.51)	N.A.	N.A.
Heroin dose change (BL-FU), iv (mg), mean (SD)	111.36 (211.73)	N.A.	N.A.
**Clinical measures**			
BDI baseline sum, mean (SD)	16.55 (7.89)	2.67 (4)	*W* = 187.5, ***P* < 0.001**
BDI follow-up sum, mean (SD)	12.72 (9.04)	3.13 (5.03)	*W* = 152.5, ***P* < 0.01**
BDI change (BL-FU), mean (SD)	3.82 (7.06)	−0.5 (3.07)	*W* = 122, *P* = 0.12
HCQ baseline sum, mean (SD)	157 (25.37)	N.A.	N.A.
HCQ follow-up sum, mean (SD)	145.62 (57.89)	N.A.	N.A.
HCQ change (BL-FU), mean (SD)	11.95 (54.52)	N.A.	N.A.

DAM = Diacetylmorphine, BDI = Beck Depression Inventory, HCQ = Heroin craving questionnaire, N.A. = not applicable, BL = Baseline, FU = Follow-up. ^a^ Baseline no and follow-up yes/no change/baseline yes and fallow-up no. ^b^ 1 missing value. ^c^ sum of intravenous dose and 0.5*per oral dose. Bold values are *P*-values below <0.05.

### Seed-to-voxel analysis

**Figure 2 Resting-state functional connectivity (rsFC) from binge/intoxication regions to the right superior frontal gyrus (SFG).**
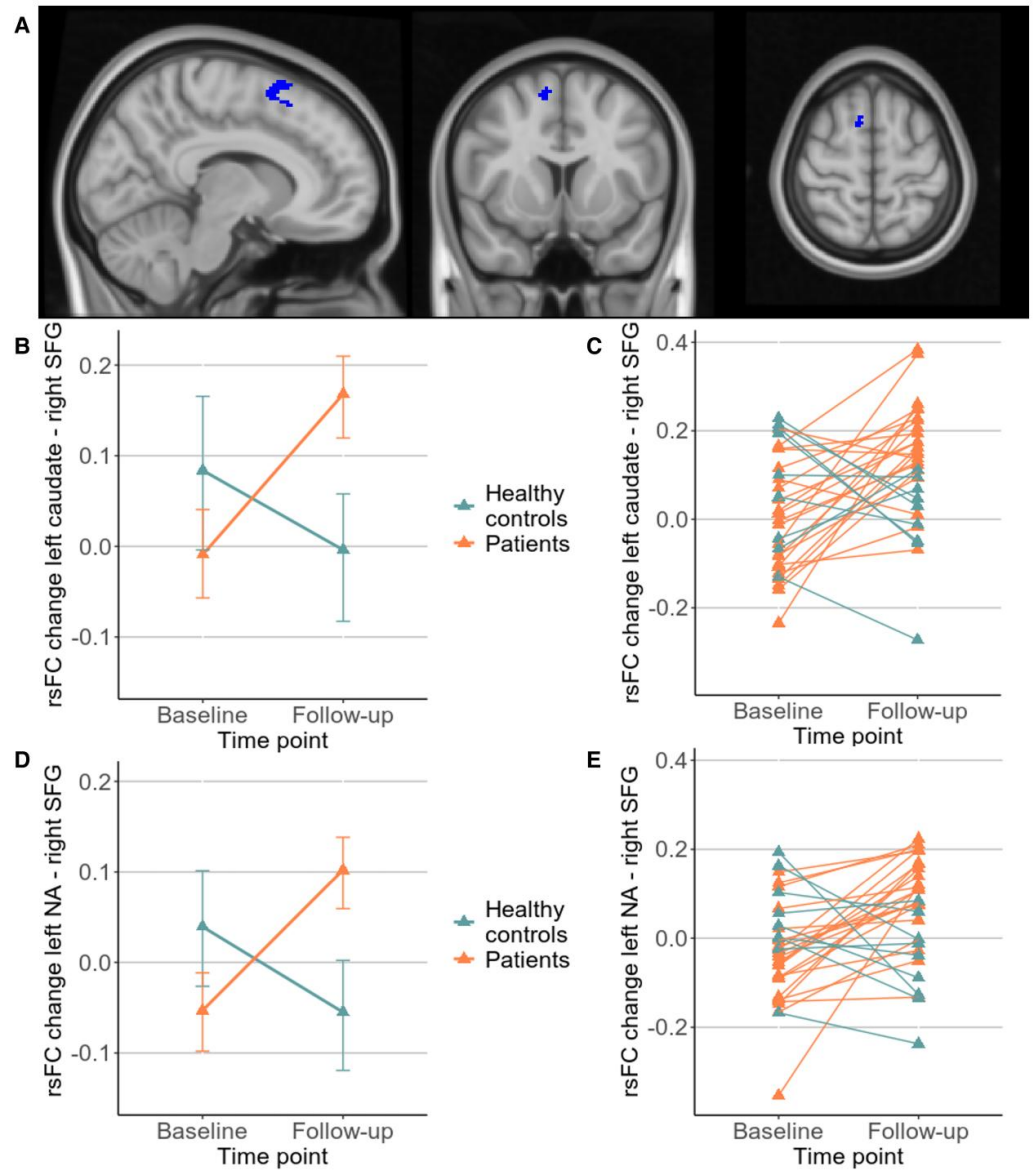
(**A**) Location of the cluster in right SFG (*x* = +10, *y* = +10, *z* = +60). Significant posthoc tests in rsFC from (**B**) left caudate (*T*(29) = 4.88, *P* < 0.001) with (**C**) individual trajectories and (**D**) left nucleus accumbens (NA) (*T*(29) = 6.69, *P* < 0.001) with (**E**) corresponding individual trajectories.

For the six regions of binge (bilateral caudate, putamen, nucleus accumbens), a significant group × time interaction was found in a cluster in the right superior frontal gyrus (SFG, *x* = +10 *y* = +10 *z* = +60, 63 voxel, Fig. 2A) extending to the juxtapositional lobule cortex. Subsequent post-hoc testing showed that the rsFC between the left nucleus accumbens and right SFG (beta = 8.6, *T*(29) = 6.69, *P* < 0.001) as well as between the left caudate and right SFG (beta = 6.03, *T*(29) = 4.88, *P* < 0.001) increased in the patient group and decreased in healthy controls (Fig. 2B and D). Fig. 2C and E shows individual trajectories of rsFC in these two connections. A significant time effect but no interaction was found in the connection originating from the right caudate (*T*(29) = 2.36, *P* < 0.05), indicating an increase of connectivity in both groups. Other time effects and all group effects were not significant. In the additional analyses with the two subsamples of patients, the cluster in the SFG was detected in both subsamples after adapting the cluster size threshold to *P*-uncorrected < 0.05 (see Supplementary Tables 1 and 2 for details on the detected clusters).

### Associations of rsFC and clinical measures

Exploratory analyses showed that the duration of DAM treatment explained variance of changes in rsFC from the left nucleus accumbens to right SFG (*F*(1,19) = 12.00, *P* < 0.01); that is the longer the DAM treatment the smaller rsFC changes (increase) (*r* = 0.63, *P* < 0.01, Fig. 3). One patient was excluded in the correlation due to missing data in the duration of DAM treatment variable. When removing a bivariate outlier (MD = 7.51), the correlation remained significant (*r* = 0.58, *P* < 0.05). Interestingly, age was not correlated with the duration of the DAM treatment (*r* = 0.16, *P* = 0.49); partial correlation including age did not affect the association of rsFC changes and the duration of DAM.

**Figure 3 Significant positive correlation (r = 0.63, P < 0.01) between resting-state functional connectivity (rsFC) changes in left nucleus accumbens (NA) to right superior frontal gyrus (SFG) and duration of DAM treatment (in years) in patients.**
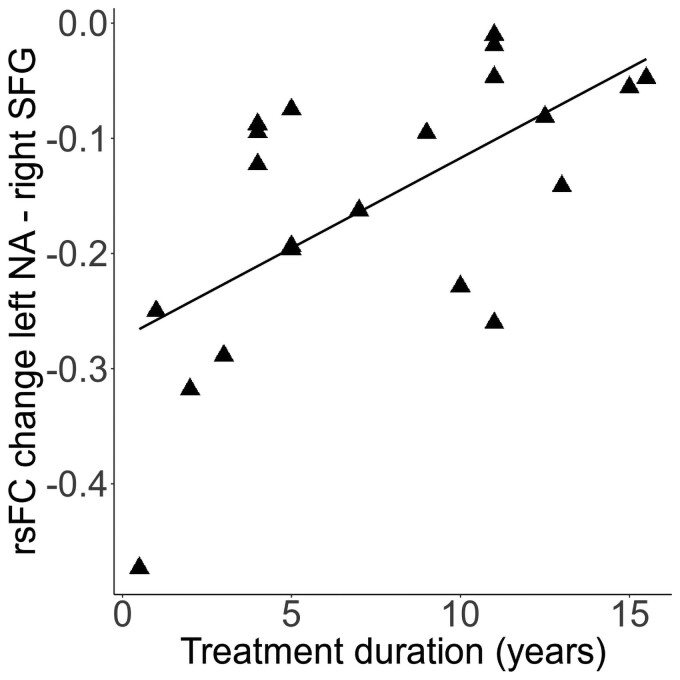


Further heroin-related measures such as age of first use, duration of dependence at baseline and doses of heroin at baseline and follow-up were not related to changes in rsFC.

Correlations and linear models including baseline, follow-up or change scores of BDI-II and the HCQ scores showed no significant links between rsFC and craving or depressive symptoms.

## Discussion

This study investigated long-term effects of DAM treatment on striatal rsFC in chronic OUD patients. Increases in rsFC were found in the left nucleus accumbens and left caudate both connecting to the right SFG in OUD patients compared to healthy controls. The strength of rsFC changes in the left nucleus accumbens was positively related to the duration of the DAM treatment, indicating greater effects at the beginning of the therapy.

These results are in line with previous findings that point out the relevance of fronto-striatal connections in addiction although we could not confirm our specific hypothesis concerning the prefrontal cortex but found a significant cluster in the right SFG. Functionally related activity between the dorsal caudate and frontal regions including the superior frontal gyrus have been detected in a seminal work in healthy participants.^38^ An early study in OUD patients found increased rsFC between the nucleus accumbens and orbitofrontal cortex compared to healthy controls.^39^ A PET study with cocaine users further showed that the regulation of these specific fronto-accumbal connections might be crucial for the ability to inhibit craving.^40^ They postulate that strengthening the regulation of this connection may therefore be a possible new treatment target for patients with addiction. However, we could not find a link between fronto-striatal connectivity increases and craving. Possible reasons could be that the substance of addiction and the applied methods were not the same than ours.

In patients with prescribed opioid-dependence, decreases in rsFC originating from the amygdala, anterior insula and nucleus accumbens have been found in comparison to healthy subjects in a cross-sectional study.^19^ More specifically, rsFC reductions in connections from the nucleus accumbens to subcortical and cortical regions such as the orbitofrontal cortex were detected. In addition, the connectivity strengths from the nucleus accumbens to the orbitofrontal cortex and also to the anterior cingulate were positively related to the duration of the prescription opioid exposure; that is subjects with the shortest duration had lowest rsFC in comparison to healthy controls. The authors postulate that this effect might be due to specific initial effects of prescription opioid exposure.^19^ However, also in long-term abstinent heroin-dependent subjects, is has been shown that the nucleus accumbens functional network is still dysfunctional.^41^ Increased rsFC has been detected between the nucleus accumbens and the right ventromedial prefrontal cortex and decreased connectivity between the nucleus accumbens and the supplementary motor area, left putamen and left precuneous compared to a sample of healthy controls. This is in line with our results that a normalization of fronto-striatal rsFC in heroin-dependent subjects receiving DAM therapy is not present.

The role of the SFG in addiction has not been elucidated fully yet. A PET study in polysubstance users showed increased glucose utilization in the SFG and other regions such as middle temporal gyrus, insula and a strongest effect in the orbitofrontal cortex after a placebo injection in comparison to healthy controls.^42^ The authors suggest that the effect in these cortical regions may be related to motivational aspects, meaning that a placebo injection activates a motivation-related circuit. Generally, it is assumed that the right SFG is specifically important for variations in action restriction and control of impulsive responses.^43^ In a study with cocaine users using a response inhibition task, healthy controls showed higher activation in the right SFG and the right supplementary motor cortex compared to cocaine users during correct inhibition events.^44^ However, the activity in these regions was not linked to the behavioural performance (correct inhibitions). It has also been postulated that the SFG consists of functional subregions with distinct connection patterns.^45^ In our study, the cluster was located closely to the juxtapositional lobule cortex (formerly supplementary motor cortex), which is related to different motor functions and important for linking cognition to action.^46^ Reduced grey matter volume in the prefrontal cortex, supplementary motor cortex and the cingulate cortex has been found in heroin-dependent subjects compared to healthy controls, suggesting a role in the neuropathology of heroin dependence.^47^ However, we were not able to link our rsFC effects to any behavioural outcome as suggested with our a priori hypothesis, postulating relations of rsFC changes with craving. This might be due to the timing of the craving assessment shortly after the DAM intake showing generally low and stable craving values at baseline and follow-up (mean per item baseline: 3.49, follow-up: 3.24 = neutral answer). Depressive symptoms decreased over the nine-year period, an effect reflecting clinical beneficial effects of the DAM treatment, but it was not linked to rsFC changes. Furthermore, we could not find an association between rsFC changes and the duration of the opioid use disorder or the DAM dose as hypothesized. However, we found an association with the duration of the DAM treatment showing that the DAM affects rsFC stronger at the beginning of the treatment and lowers over time. Also, we could show that this association was unrelated to the age of the participants. This could be an indication that a habituation effect occurs over time with strongest effects of DAM at the beginning of the treatment. Based on our findings, age and the duration of the opioid use seem not to play an essential role in this rsFC effect, supporting the applicability of the DAM treatment independently of these factors.

Some limitations of the study need to be addressed. We could not find any relations of fronto-striatal connectivity changes with behavioural measures such as craving, and data were not available for cognition-related behaviours. As the detected regions are highly linked to cognitive control and response inhibition in healthy controls and patients with addiction,^16, 38, 48^ future studies may include further behavioural and cognitive measures to disentangle the clinical relevance of the findings. Moreover, some patients changed route of administration of DAM from intravenous to oral. These two application forms differ in their bioavailability and pharmacokinetics.^49^ Oral DAM administration is a safe and effective application mode^50^ but it does not trigger a rush. We were able to include data from a healthy control sample to exclude general aging effects. However, the study groups were not balanced and relatively small; the control group consisted of only nine subjects. Several participants of the original study were lost to follow-up. Nevertheless, it is a strength of the study that we were able to include 22 of the original 29 DAM patients, a population very difficult to follow-up for longer periods. With our rather small sample, the reproducibility of our results may be questioned^51^ although we were able to reproduce our results with adapted threshold in split samples. Generally, the reliability and reproducibility of actual neuroimaging analyses using MRI data are challenging.^52^ As subcortical seeds are associated with low reliability,^53^ our interest in seeds related to the binge state of addiction and the long test–retest interval may be sources of reduced reliability in comparison to shorter test–retest intervals and cortical seeds.^54^ Furthermore, the data acquisition of 5 min were relatively short. With short scan durations discomfort in the scanner and movement can be decreased, but it reduces the reliability of the scan^55^ and limits corrections in the preprocessing such as the exclusion of first scans. The results should be interpreted with these caveats in mind.^51^

To conclude, this study showed first evidence that prolonged DAM treatment strengthens rsFC in fronto-striatal connections, an effect that diminished after longer duration of the therapy. Further research could deepen the understanding of this effect and its clinical relevance in chronic heroin-dependent patients such as cognitive and reward-related effects of prolonged DAM treatment.

## Supplementary Material

fcac275_Supplementary_Data

## Abbreviations

ALFF: amplitude of low-frequency fluctuation
BDI-II: Beck depression inventory II
DAM: diacetylmorphine
GLM: general linear model
HCQ: heroin craving questionnaire
MD: Mahalanobis’ distance
MNI: Montreal Neurological Institute
MPRAGE: magnetization prepared rapid acquisition gradient
OAT: opioid-agonist treatment
rsFC: resting-state functional connectivity
SFG: superior frontal gyrus
