# Supplementary material for: Striatal resting-state connectivity after long-term diacetylmorphine treatment in opioid-dependent patients
Source: Brain Commun. 2022 Oct 26;4(6):fcac275. doi: 10.1093/braincomms/fcac275 (PMC9642101; doi:10.1093/braincomms/fcac275)
Supplement: fcac275_Supplementary_Data [file fcac275_supplementary_data.zip › Original_submission_manuscript.pdf]

OXFORD UNIVERSITY PRESS | Brain Communications

Striatal resting-state connectivity after long-term diacetylmorphine treatment in opioid-dependent patients

Journal:	*Brain Communications*
Manuscript ID	BRAINCOM-2022-089
Manuscript Type:	Original Article
Date Submitted by the Author:	28-Feb-2022
Complete List of Authors:	Schaub, Anna-Chiara; University Psychiatric Clinics Basel,<br>Vogel, Marc; University Psychiatric Clinics Basel<br>Baumgartner, Sophie; University Psychiatric Clinics Basel<br>Lang, Undine; University of Basel, Department of Psychiatry (UPK)<br>Borgwardt, Stefan; University Hospital Schleswig Holstein Lubeck Branch Clinic of Psychiatry and Psychotherapy<br>Schmidt, André; University of Basel, Department of Psychiatry (UPK)<br>Walter, Marc; Psychiatrische Dienste Aargau AG
Keywords:	diacetylmorphine, heroin maintenance, resting-state functional MRI, long-term effects, striatum

SCHOLARONE™
Manuscripts

Striatal resting-state connectivity after long-term diacetylmorphine treatment in opioid-dependent patients

Anna-Chiara Schaub,[1] Marc Vogel,[1] Sophie Baumgartner,[1] Undine E. Lang,[1] Stefan Borgwardt,[2] André Schmidt[1,*] and Marc Walter[1,*]

1 University of Basel, Department of Psychiatry (UPK), 4002 Basel, Switzerland

2 University of Lübeck, Department of Psychiatry and Psychotherapy, 23562 Lübeck, Germany

* shared last authors

Correspondence to: Anna-Chiara Schaub

Full address: Wilhelm-Klein Strasse 27, 4002 Basel, Switzerland

E-mail: annachiara.schaub@unibas.ch

Running title: Neural long-term effects of DAM therapy

For Review Only

Abstract

New treatment approaches for opioid-dependent patients include injectable opioid agonist treatment with diacetylmorphine (DAM). While evidence has shown beneficial clinical effects of DAM, it is still not clear how long-term DAM affects the brain and whether functional brain changes are accompanied by clinical improvements. Therefore, this prospective case-control study focuses on long-term effects of DAM on resting-state functional connectivity.

We included opioid-dependent patients (N=22, age range 33-58, 16 males) treated with DAM and healthy controls (N=9, age range 27-55, 5 males) that underwent two MRI assessments approximately nine years apart. For the patients, the assessments took part shortly after the DAM intake to be able to explore changes in resting-state functional connectivity in brain regions related to the stage of binge and intoxication (caudate, putamen, nucleus accumbens). A cluster in the right superior frontal gyrus was detected, showing over nine years an increase in functional connectivity originating from the left caudate and the left accumbens in patients but not in healthy controls. These connectivity changes in patients were related to the duration of the DAM treatment at the follow-up, indicating smaller increases in functional connectivity with longer DAM treatment ($r=0.63$, $p<.01$).

These results suggest that long-term DAM treatment in opioid-dependent patients increases fronto-striatal connections, an effect that is linked to the duration of the DAM treatment. Future research needs to further address the wide-ranging effects of DAM on brain functioning and deepen the understanding of their clinical relevance.

Keywords: diacetylmorphine; heroin maintenance; long-term effects; resting-state functional MRI; striatum

Abbreviations: ALFF = amplitude of low frequency fluctuation; BDI-II = Beck depression inventory II; DAM = diacetylmorphine; GLM = general linear model; HCQ = heroin craving questionnaire; MD = Mahalanobis' distance; MNI = Montreal Neurological Institute; MPRAGE = magnetization prepared rapid acquisition gradient; OAT = opioid-agonist treatment; rsFC = resting-state functional connectivity; SFG = superior frontal gyrus

For Review Only

Introduction

The ongoing worldwide opioid crisis is of great importance as the direct burden of disability-adjusted life years is highest for opioid dependent subjects in comparison to other illicit drugs.[1] For this crisis, by its changing nature, it is challenging to find effective and adequate responses such as prevention and treatment approaches.[2] To reduce the burden of opioid use disorder (OUD), various treatments exist to date including different options of opioid-agonist treatments (OAT). As not all patients respond to these first-line treatments, diacetylmorphine (DAM) as additional treatment approach for treatment-refractory chronic heroin dependent patients was introduced.[3] DAM is a cost-effective alternative for non-responding patients to other OAT such as methadone or buprenorphine.[4] DAM has beneficial clinical effects by increasing treatment retention and reducing illicit opioid use, but necessitates close monitoring to avoid overdoses and seizures.[5-7] Studies found additional beneficial effects such as improved physical and mental health,[8,9] improved employment status[6] and reduced criminal activity even though it is not clear if this specific effect is stronger than in oral methadone.[10]

For Review Only

Regarding the underlying neural mechanisms of OUD, research sheds light on the importance of the reward system early in the development of the disease.[11] The reward system is crucially mediating the binge or intoxication stage shortly after drug intake and is as such postulated to be predominant at early stages of drug addiction.[11,12] The binge/intoxication stage involves dopaminergic pathways in the basal ganglia, whereby the ventral striatum (nucleus accumbens) plays a key role in the acute hedonic effects and the dorsal striatum (putamen and nucleus caudate) in subsequent habit formation,[13] contributing both to compulsive substance seeking.[14] Later in the course of drug development, stages of withdrawal along with negative affect and as a third stage craving or preoccupation, which imply regions such as the amygdala and prefrontal and orbitofrontal regions, respectively, motivate continued drug intake.[11] As a circular cascade, these later stages of withdrawal and craving in turn initiate new rounds of drug consumption through aberrant fronto-striatal interactions.[15,16]

OUD has been associated with structural and functional alterations in the striatum. Decreased volume of the nucleus accumbens and putamen was found in OUD patients compared to healthy controls.[17,18] Notably, volumes in nucleus accumbens and putamen were negatively related to depression severity[17] and the duration of heroin abuse.[18] However, no volumetric differences in the striatum have been reported in another study with heroin-dependent

patients.[19] A resting-state functional MRI study further showed decreased amplitude of low frequency fluctuation (ALFF) in OUD patients in the right caudate, anterior cingulate cortex and superior frontal cortex, whereas ALFF values in the right caudate were negatively associated with the duration of heroin use and daily heroin dose.[20] Furthermore, compared with non-relapsers, heroin relapsers showed increased regional homogeneity in the right caudate that was positively related to heroin relapse rates and craving responses.[21] OUD is also associated with weaker fronto-striatal resting-state functional connectivity[22] which has also been shown in other substance use disorders.[23,24] In nicotine dependent subjects, smoking and its subsequent reduction in craving are linked to increases in fronto-striatal rsFC and more precisely in the dorsolateral prefrontal cortex.[25] Interestingly, the opioid receptor antagonist naltrexone leads to lower opioid wanting by increasing fronto-striatal connectivity also affecting the dorsolateral prefrontal cortex.[26] We have previously showed that acute DAM administration still increased dorsal striatal connectivity in OUD patients, which correlated positively with the plasma level of morphine and the subjective feeling of rush.[27] These results show that even after prolonged heroin intake, the drug still releases rewarding effects reflected by increased striatal activity, although previous research had questioned the prolonged involvement of dopamine located especially in the striatum in addiction.[28] Not much evidence is available regarding long-term DAM treatment and its effects on the brain. A voxel-based morphometry analysis showed increased volume of the right caudate and reductions in the right amygdala, anterior cingulate cortex and the orbitofrontal cortex and was able to link these effects to drug-related measures such as the DAM dose.[29] However, the interpretation of the results must be done with caution since no control sample with healthy subjects was included.

For Review Only

Long-term effects of DAM are important to investigate since patients often remain in treatment for a protracted period and termination rates are low.[5,30] Beside its beneficial clinical effects, it is of great interest to understand how DAM treatment affects the brain and especially brain functioning. This study therefore focuses on effects of DAM on rsFC. We conducted a resting-state functional MRI assessment shortly after the DAM intake and compared changes over a nine-year period to changes in healthy control subjects. Based on previous research, we expected rsFC increases in OUD patients compared to healthy controls in connections originating from the striatum to the prefrontal cortex. Furthermore, we explored whether functional brain changes are linked to clinical and behavioral measures expecting a negative association with craving as its association with fronto-striatal

connections have previously been shown[31] and furthermore an association with the duration of the opioid use and DAM dose.

For Review Only

Materials and methods

This study represents a nine-year follow-up analysis of a randomized placebo-controlled, crossover trial exploring acute DAM effects in OUD patients.[27,32,33] First follow-up results on the long-term effects of DAM on brain volume changes in OUD patients are reported elsewhere.[29] Here we report findings of long-term DAM effects in OUD patients compared to general aging effects in healthy controls on striatal rsFC.

Participants

Out of 27 patients with OUD that were included in the original baseline study,[32] 22 patients completed a nine-year follow-up MRI assessment. For initial inclusion, patients had to be older than 18 years and had a history of OUD with current injectable opioid treatment (> 6 months). The dose had to be unchanged during the previous three months. Patients were excluded when they had a positive alcohol breathalyzer test and additional physical or psychiatric diseases including severe substance use disorders. Tobacco use and alcohol or drug abuse such as cocaine and cannabis led not to exclusion. Patients had at least two unsuccessful treatments for OUD and all patients were enrolled in a standardized OAT program (JANUS, University of Basel, Department of Psychiatry [Universitäre Psychiatrische Kliniken], Switzerland), which includes the prescription of DAM and psychosocial treatment. History of heroin and other illicit substance use was assessed, and behavioral measures related to heroin dependence such as duration of dependence at baseline (in years), age of first use, duration of the DAM treatment at baseline (in years) and the daily opioid dose (baseline and follow-up in mg) were recorded.

Beside OUD patients, nine out of 20 healthy controls from an initial baseline sample were included in this nine-year follow-up MRI study. Healthy controls had no psychiatric or neurological diagnoses and no family history of psychiatric illness.

The study was approved by the local ethics committee (Ethikkommission Nordwest und Zentralschweiz) according to the Declaration of Helsinki, and all participants gave written informed consent before inclusion in the study.

Study design

Patients administered DAM and underwent an MRI session 20 minutes after the intake. Depressive symptoms and craving were assessed shortly after the DAM intake using the Beck depression inventory (BDI-II)[34] and the heroin craving questionnaire (HCQ)[35], respectively. Medication and side consummation was tracked during the study interval. At the nine-years

For Review Only

follow-up assessment, the same MRI session, the BDI-II and HCQ were assessed again. Healthy controls did not receive any treatment but underwent two MRI sessions at a nine-year interval.

MRI assessment

Patients and healthy controls underwent an MRI session including structural and resting-state functional MRI sequences at the baseline and follow-up assessment using a 3T MAGNETOM VERIO scanner (SIEMENS, Erlangen, Germany) with a 12-channel radiofrequency head coil. Foam pads across the forehead were used to minimize head movement. For the 5 min resting-state sequence, subjects were instructed to lie in the scanner with eyes open, to think of nothing in particular, and not to fall asleep. We used a gradient echo planar imaging (EPI) sequence (TR=2000ms, TE=28ms, slice thickness 3.3mm, field of view 228mm, flip angle 82°, 152 volumes, voxel size 3.6x3.6x3.3mm, bandwidth of 2694 Hz/pixel) and for anatomical reference, a 3D whole-brain T1-weighted magnetization prepared rapid acquisition gradient (MPRAGE) sequence was applied (176 slices, field of view 256mm, TR=2000ms, TE=3.37ms, flip angle 8°, 1mm slice thickness, 1x1x1mm voxel size, bandwidth of 200 Hz/pixel).

For Review Only

Resting-state analysis

Functional MRI data was processed and analyzed using the CONN toolbox (19.c, http://www.nitrc.org/projects/conn),[36] an open-source Matlab/SPM-based software. For preprocessing, a default pipeline for volume-based analysis was applied including realignment and unwarping (subject motion estimation and correction), slice timing correction, outlier detection using ART-based identification, segmentation into grey and white matter and CSF, direct normalization into standard Montreal Neurological Institute (MNI) space and smoothing (gaussian kernel FWHM = 6mm) in the presented order. Denoising was done using linear regression of potential confounding effects implemented with an anatomical component-based noise correction procedure (aCompCor). It includes subject motion parameters, scrubbing,[37] noise components from white matter and cerebrospinal areas and session effects. Finally, linear detrending and temporal band-pass filtering ($0.0008 < f < 0.09$ Hz) were applied.

Seed-based functional MRI connectivity analysis

Brain regions that have been linked to the binge/intoxication stage of dependence were selected as seeds such as the dorsal striatum (bilateral caudate, putamen) and the nucleus accumbens (Figure 1).[11] Brain maps of bivariate correlation coefficients (fisher transformed) were

calculated for each subject, time point, and seed. The general linear model (GLM) was set to an 2x2 mixed ANOVA interaction comparing OUD patients and healthy controls over time from baseline to the follow-up assessment. All six seeds of the binge stage were jointly included as sources; targets were voxels covering the whole brain. Change scores of smoking (quantity at baseline - follow-up) and a score of cocaine, benzodiazepine, and cannabis use (-1: baseline no/follow-up yes, 0: no change, 1: baseline yes/fallow-up no) were demeaned across all subjects and included as covariates to avoid confounding effects. To define clusters of interest, an uncorrected threshold of $p<.001$ was applied and results were considered significant with an FDR-corrected $p<.05$ cluster-level threshold.

Behavioral and exploratory analysis

Comparisons between study groups in behavioral and clinical measures were conducted using chi-squared tests, two sample t-tests and non-parametric Wilcoxon signed rank tests.
Exploratory analyses were added to investigate if behavioral (BDI-II, HCQ) and heroin-related measures (duration of DAM treatment, duration of heroin dependence, age of first use, daily opioid dose at baseline and follow-up) were related to significant rsFC changes (baseline – follow-up). Therefore, linear models were set including different covariates (age and gender) and an analysis of variance (ANOVA, type III) was run over the model with the best fit. In addition, spearman correlations were conducted in the heroin group including change scores of behavioral measures as mentioned above and changes in rsFC. Possible bivariate outliers were detected using mahalanobis' distance (MD) and participants were removed from correlations when above the cutoff (chi-square distance with $p=.95$, $df=2$). All statistical analyses were carried out using R.

Data availability

Anonymized data are available upon reasonable request to the corresponding authors.

For Review Only

Results

Participants

OUD patients and healthy controls did not differ significantly in age and gender, however, differences in clinical and behavioral measures occurred such as in education, smoking, cocaine use, and depressive symptoms (Table 1). In OUD patients, the DAM dose and delivery form changed in some patients but not all; some patients switched from intravenous application to oral and two patients did no longer receive any DAM at the follow-up. The mean intravenous dose dropped significantly at the follow-up ($t(21)=2.47$, $p<0.05$), but when combining intravenous and oral doses at follow-up, the dose was not significantly different to the initial dose at baseline ($t(21)=-0.11$, $p=0.9$). Depressive symptoms in the patients decreased over the study period ($V=163.5$, $p<0.5$), while craving (HCQ) did not change significantly ($V=139.5$, $p=0.41$). Depressive symptoms in healthy controls did not change over time ($V=9$, $p=.44$).

Seed-to-voxel analysis

For the six regions of binge (bilateral caudate, putamen, nucleus accumbens), a significant group x time interaction was found in a cluster in the right superior frontal gyrus (SFG, x=+10 y=+10 z=+60, 63 voxel, Figure 2A) extending to the juxtapositional lobule cortex. Subsequent post-hoc testing showed that the rsFC between the left nucleus accumbens and right SFG as well as between the left caudate and right SFG increased in the patient group and decreased in healthy controls (Figure 2B and 2D). Figure 2C and 2E show individual trajectories of rsFC in these two connections. A significant time effect but no interaction was found in the connection originating from the right caudate, indicating an increase of connectivity in both groups. Other time effects and all group effects were not significant.

Associations of rsFC and clinical measures

Exploratory analyses showed that the duration of DAM treatment explained variance of changes in rsFC from the left nucleus accumbens to right SFG ($F(1,19)=12.00$, $p<.01$); that is the longer the DAM treatment the smaller rsFC changes (increase) ($r=0.63$, $p<.01$, Figure 3). One patient was excluded in the correlation due to missing data in the duration of DAM treatment variable. When removing a bivariate outlier (MD=7.51), the correlation remained significant ($r=0.58$, $p<.05$). Interestingly, age was not correlated with the duration of the DAM treatment ($r=0.16$, $p=.49$); partial correlation including age did not affect the association of rsFC changes and the duration of DAM.

For Review Only

Further heroin related measures such as age of first use, duration of dependence at baseline and doses of heroin at baseline and follow-up were not related to changes in rsFC.

Correlations and linear models including baseline, follow-up or change scores of BDI-II and the HCQ scores showed no significant links between rsFC and craving or depressive symptoms.

For Review Only

Discussion

This study investigated long-term effects of DAM treatment on striatal rsFC in chronic OUD patients. Increases in rsFC were found in the left nucleus accumbens and left caudate both connecting to the right SFG in OUD patients compared to healthy controls. The strength of rsFC changes in the left nucleus accumbens was positively related to the duration of the DAM treatment, indicating greater effects at the beginning of the therapy.

These results are in line with previous findings that point out the relevance of fronto-striatal connections in addiction although we could not confirm our specific hypothesis concerning the prefrontal cortex but found a significant cluster in the right SFG. In particular, an early study in OUD patients found increased rsFC between the nucleus accumbens and orbitofrontal cortex compared to healthy controls.[38] A PET study with cocaine users further showed that the regulation of these specific fronto-accumbal connections might be crucial for the ability to inhibit craving.[39] They postulate that strengthening the regulation of this connection may therefore be a possible new treatment target for patients with addiction. However, we could not find a link between fronto-striatal connectivity increases and craving. Possible reasons could be that the substance of addiction and the applied methods were not the same than ours.

In patients with prescribed opioid-dependence, decreases in rsFC originating from the amygdala, anterior insula and nucleus accumbens have been found in comparison to healthy subjects in a cross-sectional study.[19] More specifically, rsFC reductions in connections from the nucleus accumbens to subcortical and cortical regions such as the orbitofrontal cortex were detected. In addition, the connectivity strengths from the nucleus accumbens to the orbitofrontal cortex and also to the anterior cingulate were positively related to the duration of the prescription opioid exposure; that is subjects with the shortest duration had lowest rsFC in comparison to healthy controls. The authors postulate that this effect might be due to specific initial effects of prescription opioid exposure.[19] However, also in long-term abstinent heroin-dependent subjects, is has been shown that the nucleus accumbens functional network is still dysfunctional.[40] Increased rsFC has been detected between the nucleus accumbens and the right ventromedial prefrontal cortex and decreased connectivity between the nucleus accumbens and the supplementary motor area, left putamen and left precuneous compared to a sample of healthy controls. This is in line with our results that a normalization of fronto-striatal rsFC in heroin-dependent subjects receiving DAM therapy is not present.

For Review Only

The role of the SFG in addiction has not been elucidated fully yet. A PET study in polysubstance users showed increased glucose utilization in the SFG and other regions such as middle temporal gyrus, insula and a strongest effect in the orbitofrontal cortex after a placebo injection in comparison to healthy controls.[41] The authors suggest that the effect in these cortical regions may be related to motivational aspects, meaning that a placebo injection activates a motivation related circuit. Generally, it is assumed that the right SFG is specifically important for variations in action restriction and control of impulsive responses.[42] In a study with cocaine users using a response inhibition task, healthy controls showed higher activation in the right SFG and the right supplementary motor cortex compared to cocaine users during correct inhibition events.[43] However, the activity in these regions was not linked to the behavioral performance (correct inhibitions). It has also been postulated that the SFG consists of functional subregions with distinct connection patterns.[44] In our study, the cluster was located closely to the juxtapositional lobule cortex (formerly supplementary motor cortex), which is related to different motor functions and important for linking cognition to action.[45] Reduced gray matter volume in the prefrontal cortex, supplementary motor cortex and the cingulate cortex has been found in heroin dependent subjects compared to healthy controls, suggesting a role in the neuropathology of heroin dependence.[46] However, we were not able to link our rsFC effects to any behavioral outcome as suggested with our a priori hypothesis, postulating relations of rsFC changes with craving. This might be due to the timing of the craving assessment shortly after the DAM intake showing generally low and stable craving values at baseline and follow-up (mean per item baseline: 3.49, follow-up: 3.24 = neutral answer). Depressive symptoms decreased over the nine-year period, an effect reflecting clinical beneficial effects of the DAM treatment, but it was not linked to rsFC changes. Furthermore, we could not find an association between rsFC changes and the duration of the opioid use disorder or the DAM dose as hypothesized. However, we found an association with the duration of the DAM treatment showing that the DAM affects rsFC stronger at the beginning of the treatment and lowers over time. Also, we could show that this association was unrelated to the age of the participants. This could be an indication that a habituation effect occurs over time with strongest effects of DAM at the beginning of the treatment. Based on our findings, age and the duration of the opioid use seem not to play an essential role in this rsFC effect, supporting the applicability of the DAM treatment independently of these factors.

For Review Only

Some limitations of the study need to be addressed. We were able to include data from a healthy control sample to exclude general aging effects. However, the study groups were not balanced; the control group consisted of only nine subjects. Several participants of the original study were lost to follow-up. However, it is a strength of the study that we were able to include 22 of the original 29 DAM patients, a population very difficult to follow-up for longer periods. Furthermore, the data acquisition of 5 min was relatively short. With short scan durations discomfort in the scanner and movement can be decreased, but it reduces the reliability of the scan[47] and limits corrections in the preprocessing such as the exclusion of first scans. In our study, the assessment of clinical and behavioral measures was limited; further assessments such as impulsivity[48] or cognitive skills would have been interesting. Moreover, some patients changed route of administration of DAM from intravenous to oral. These two application forms differ in their bioavailability and pharmacokinetics.[49] Oral DAM administration is a safe and effective application mode[50] but it does not trigger a rush.

To conclude, this study showed first evidence that prolonged DAM treatment strengthens rsFC in fronto-striatal connections, an effect that diminished after longer duration of the therapy. Further research could deepen the understanding of this effect and its clinical relevance in chronic heroin dependent patients such as cognitive and reward related effects of prolonged DAM treatment.

Funding

The study was supported by the Freiwillige Akademische Gesellschaft (FAG) Basel and the Stiftung zur Förderung der gastroenterologischen und allgemeinen klinischen Forschung sowie der medizinischen Bildauswertung (AS).

Competing interests

The authors report no competing interests.

For Review Only

References

1. Degenhardt L, Whiteford HA, Ferrari AJ, et al. Global burden of disease attributable to illicit drug use and dependence: findings from the Global Burden of Disease Study 2010. *Lancet*. Nov 2013;382(9904):1564-1574. doi:10.1016/s0140-6736(13)61530-5
2. Volkow ND, Blanco C. The changing opioid crisis: development, challenges and opportunities. *Molecular Psychiatry*. Jan 2021;26(1):218-233. doi:10.1038/s41380-020-0661-4
3. Uchtenhagen AA. Heroin maintenance treatment: From idea to research to practice. *Drug and Alcohol Review*. Mar 2011;30(2):130-137. doi:10.1111/j.1465-3362.2010.00266.x
4. Mielau J, Vogel M, Gutwinski S, Mick I. New Approaches in Drug Dependence: Opioids. *Current Addiction Reports*. Jun 2021;8(2):298-305. doi:10.1007/s40429-021-00373-9
5. Oviedo-Joekes E, Brissette S, Marsh DC, et al. Diacetylmorphine versus Methadone for the Treatment of Opioid Addiction. Article. *New England Journal of Medicine*. Aug 2009;361(8):777-786. doi:10.1056/NEJMoa0810635
6. Haasen C, Verthein U, Degkwitz P, Berger J, Krausz M, Naber D. Heroin-assisted treatment for opioid dependence - Randomised controlled trial. *British Journal of Psychiatry*. Jul 2007;191:55-62. doi:10.1192/bjp.bp.106.026112
7. Strang J, Groshkova T, Uchtenhagen A, et al. Heroin on trial: systematic review and meta-analysis of randomised trials of diamorphine-prescribing as treatment for refractory heroin addiction. *British Journal of Psychiatry*. Jul 2015;207(1):5-14. doi:10.1192/bjp.bp.114.149195
8. Demaret I, Quertemont E, Litran G, et al. Efficacy of Heroin-Assisted Treatment in Belgium: A Randomised Controlled Trial. *European Addiction Research*. 2015;21(4):179-187. doi:10.1159/000369337
9. Moazen-Zadeh E, Ziafat K, Yazdani K, et al. Impact of opioid agonist treatment on mental health in patients with opioid use disorder: a systematic review and network meta-analysis of randomized clinical trials. *American Journal of Drug and Alcohol Abuse*. May 2021;47(3):280-304. doi:10.1080/00952990.2021.1887202
10. Smart R, Reuter P. Does heroin-assisted treatment reduce crime? A review of randomized-controlled trials. *Addiction*. 2021;doi:10.1111/add.15601
11. Koob GF, Volkow ND. Neurocircuitry of Addiction. *Neuropsychopharmacology*. Jan 2010;35(1):217-238. doi:10.1038/npp.2009.110
12. Koob GF, Volkow ND. Neurobiology of addiction: a neurocircuitry analysis. *Lancet Psychiatry*. Aug 2016;3(8):760-773. doi:10.1016/S2215-0366(16)00104-8
13. Hodebourg R, Murray JE, Fouyssac M, Puaud M, Everitt BJ, Belin D. Heroin seeking becomes dependent on dorsal striatal dopaminergic mechanisms and can be decreased by N-acetylcysteine. *European Journal of Neuroscience*. Aug 2019;50(3):2036-2044. doi:10.1111/ejn.13894
14. Everitt BJ, Belin D, Economidou D, Pelloux Y, Dalley JW, Robbins TW. Neural mechanisms underlying the vulnerability to develop compulsive drug-seeking habits and addiction. *Philosophical Transactions of the Royal Society B-Biological Sciences*. Oct 2008;363(1507):3125-3135. doi:10.1098/rstb.2008.0089
15. Volkow ND, Morales M. The Brain on Drugs: From Reward to Addiction. *Cell*. Aug 2015;162(4):712-725. doi:10.1016/j.cell.2015.07.046
16. Morein-Zamir S, Robbins TW. Fronto-striatal circuits in response-inhibition: Relevance to addiction. *Brain Research*. Dec 2015;1628:117-129. doi:10.1016/j.brainres.2014.09.012

For Review Only

17. Seifert CL, Magon S, Sprenger T, et al. Reduced volume of the nucleus accumbens in heroin addiction. *European Archives of Psychiatry and Clinical Neuroscience*. Dec 2015;265(8):637-645. doi:10.1007/s00406-014-0564-y

18. Keihani A, Ekhtiari H, Batouli SAH, et al. Lower Gray Matter Density in the Anterior Cingulate Cortex and Putamen Can Be Traceable in Chronic Heroin Dependents After Over Three Months of Successful Abstinence. *Iranian Journal of Radiology*. Jul 2017;14(3)e41858. doi:10.5812/iranjradiol.41858

19. Upadhyay J, Maleki N, Potter J, et al. Alterations in brain structure and functional connectivity in prescription opioid-dependent patients. *Brain*. Jul 2010;133:2098-2114. doi:10.1093/brain/awq138

20. Wang YR, Zhu J, Li Q, et al. Altered Fronto-Striatal and Fronto-Cerebellar Circuits in Heroin-Dependent Individuals: A Resting-State fMRI Study. *Plos One*. Mar 2013;8(3)e58098. doi:10.1371/journal.pone.0058098

21. Chang HF, Li W, Li Q, et al. Regional homogeneity changes between heroin relapse and non-relapse patients under methadone maintenance treatment: a resting-state fMRI study. *Bmc Neurology*. Aug 2016;16145. doi:10.1186/s12883-016-0659-3

22. McConnell PA, Garland EL, Zubieta JK, Newman-Norlund R, Powers S, Froeliger B. Impaired frontostriatal functional connectivity among chronic opioid using pain patients is associated with dysregulated affect. *Addiction Biology*. Mar 2020;25(2)doi:10.1111/adb.12743

23. Motzkin JC, Baskin-Sommers A, Newman JP, Kiehl KA, Koenigs M. Neural Correlates of Substance Abuse: Reduced Functional Connectivity Between Areas Underlying Reward and Cognitive Control. *Human Brain Mapping*. Sep 2014;35(9):4282-4292. doi:10.1002/hbm.22474

24. Hong LE, Gu H, Yang Y, et al. Association of Nicotine Addiction and Nicotine's Actions With Separate Cingulate Cortex Functional Circuits. *Archives of General Psychiatry*. Apr 2009;66(4):431-441. doi:10.1001/archgenpsychiatry.2009.2

25. Franklin TR, Jagannathan K, Spilka NH, et al. Smoking-induced craving relief relates to increased DLPFC-striatal coupling in nicotine-dependent women. *Drug and Alcohol Dependence*. Apr 2021;221108593. doi:10.1016/j.drugalcdep.2021.108593

26. Soutschek A, Weber SC, Kahnt T, Quednow BB, Tobler PN. Opioid antagonism modulates wanting-related frontostriatal connectivity. *Elife*. Nov 2021;10e71077. doi:10.7554/eLife.71077

27. Schmidt A, Denier N, Magon S, et al. Increased functional connectivity in the resting-state basal ganglia network after acute heroin substitution. *Translational Psychiatry*. Mar 2015;5e533. doi:10.1038/tp.2015.28

28. Volkow ND, Fowler JS, Wang GJ, Swanson JM. Dopamine in drug abuse and addiction: results from imaging studies and treatment implications. *Molecular Psychiatry*. Jun 2004;9(6):557-569. doi:10.1038/sj.mp.4001507

29. Schmidt A, Vogel M, Baumgartner S, et al. Brain volume changes after long-term injectable opioid treatment: A longitudinal voxel-based morphometry study. Article; Early Access. *Addiction Biology*. 2020:9. e12970. doi:10.1111/adb.12970

30. Blanken P, Hendriks VM, van Ree JM, van den Brink W. Outcome of long-term heroin-assisted treatment offered to chronic, treatment-resistant heroin addicts in the Netherlands. *Addiction*. Feb 2010;105(2):300-308. doi:10.1111/j.1360-0443.2009.02754.x

31. Kober H, Mende-Siedlecki P, Kross EF, et al. Prefrontal-striatal pathway underlies cognitive regulation of craving. *Proceedings of the National Academy of Sciences of the United States of America*. Aug 2010;107(33):14811-14816. doi:10.1073/pnas.1007779107

32. Walter M, Denier N, Gerber H, et al. Orbitofrontal response to drug-related stimuli after heroin administration. *Addiction Biology*. May 2015;20(3):570-579. doi:10.1111/adb.12145

For Review Only

33. Schmidt A, Borgwardt S, Gerber H, et al. Acute Effects of Heroin on Negative Emotional Processing: Relation of Amygdala Activity and Stress-Related Responses. *Biological Psychiatry*. Aug 2014;76(4):289-296. doi:10.1016/j.biopsych.2013.10.019
34. Beck AT, Steer RA, Brown GK. *Beck depression inventory (BDI-II)*. vol 10. Pearson; 1996.
35. Tiffany ST. Cognitive concepts of craving. *Alcohol Research & Health*. 1999;23(3):215-224.
36. Whitfield-Gabrieli S, Nieto-Castanon A. Conn: a functional connectivity toolbox for correlated and anticorrelated brain networks. *Brain connectivity*. 2012;2(3):125-141.
37. Power JD, Mitra A, Laumann TO, Snyder AZ, Schlaggar BL, Petersen SE. Methods to detect, characterize, and remove motion artifact in resting state fMRI. *Neuroimage*. Jan 2014;84:320-341. doi:10.1016/j.neuroimage.2013.08.048
38. Ma N, Liu Y, Li N, et al. Addiction related alteration in resting-state brain connectivity. *Neuroimage*. Jan 2010;49(1):738-744. doi:10.1016/j.neuroimage.2009.08.037
39. Volkow ND, Fowler JS, Wang GJ, et al. Cognitive control of drug craving inhibits brain reward regions in cocaine abusers. *Neuroimage*. Feb 2010;49(3):2536-2543. doi:10.1016/j.neuroimage.2009.10.088
40. Zou F, Wu XH, Zhai TY, et al. Abnormal Resting-State Functional Connectivity of the Nucleus Accumbens in Multi-year Abstinent Heroin Addicts. *Journal of Neuroscience Research*. Nov 2015;93(11):1693-1702. doi:10.1002/jnr.23608
41. London ED, Ernst M, Grant S, Bonson K, Weinstein A. Orbitofrontal cortex and human drug abuse: Functional imaging. *Cerebral Cortex*. Mar 2000;10(3):334-342. doi:10.1093/cercor/10.3.334
42. Hu S, Ide JS, Zhang S, Li CSR. The Right Superior Frontal Gyrus and Individual Variation in Proactive Control of Impulsive Response. *Journal of Neuroscience*. Dec 2016;36(50):12688-12696. doi:10.1523/jneurosci.1175-16.2016
43. Hester R, Garavan H. Executive dysfunction in cocaine addiction: Evidence for discordant frontal, cingulate, and cerebellar activity. *Journal of Neuroscience*. Dec 2004;24(49):11017-11022. doi:10.1523/jneurosci.3321-04.2004
44. Li W, Qin W, Liu HG, et al. Subregions of the human superior frontal gyrus and their connections. *Neuroimage*. Sep 2013;78:46-58. doi:10.1016/j.neuroimage.2013.04.011
45. Nachev P, Kennard C, Husain M. Functional role of the supplementary and pre-supplementary motor areas. *Nature Reviews Neuroscience*. Nov 2008;9(11):856-869. doi:10.1038/nrn2478
46. Liu HH, Hao YH, Kaneko Y, et al. Frontal and cingulate gray matter volume reduction in heroin dependence: Optimized voxel-based morphometry. *Psychiatry and Clinical Neurosciences*. 2009;63(4):563-568. doi:10.1111/j.1440-1819.2009.01989.x
47. Birn RM, Molloy EK, Patriat R, et al. The effect of scan length on the reliability of resting-state fMRI connectivity estimates. *Neuroimage*. Dec 2013;83:550-558. doi:10.1016/j.neuroimage.2013.05.099
48. Wang SC, Zhang M, Liu S, et al. Impulsivity in heroin-dependent individuals: structural and functional abnormalities within frontostriatal circuits. *Brain Imaging and Behavior*. Oct 2021;15(5):2454-2463. doi:10.1007/s11682-020-00445-w
49. Girardin F, Rentsch KM, Schwab MA, et al. Pharmacokinetics of high doses of intramuscular and oral heroin in narcotic addicts. *Clinical Pharmacology & Therapeutics*. Oct 2003;74(4):341-352. doi:10.1016/s0009-9236(03)00199-1
50. Frick U, Rehm J, Kovacic S, Ammann J, Uchtenhagen A. A prospective cohort study on orally administered heroin substitution for severely addicted opioid users. *Addiction*. Nov 2006;101(11):1631-1639. doi:10.1111/j.1360-0443.2006.01569.x

For Review Only

Figure legends

Figure 1. Seed regions of seed-to-voxel analysis for binge/intoxication stage. Binge regions consisted of the bilateral caudate (green), putamen (yellow) and nucleus accumbens (blue) according to the Harvard-Oxford subcortical structural atlas as implemented in conn.

Figure 2. Resting-state functional connectivity (rsFC) from binge/intoxication regions to the right superior frontal gyrus (SFG). (A) Location of the cluster in right SFG (x=+10, y=+10, z=+60). Significant group*time interactions in rsFC from **(B)** left caudate with **(C)** individual trajectories and **(D)** left nucleus accumbens (NA) with **(E)** corresponding individual trajectories.

Figure 3. Significant positive correlation (r=0.63, p<.01) between resting-state functional connectivity (rsFC) changes in left nucleus accumbens (NA) to right superior frontal gyrus (SFG) and duration of DAM treatment (in years) in OUD patients.

For Review Only

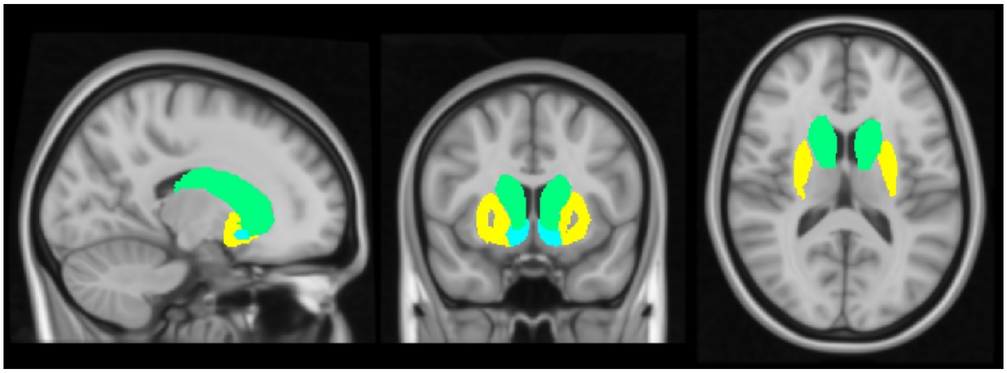

Figure 1. Seed regions of seed-to-voxel analysis for binge/intoxication stage. Binge regions consisted of the bilateral caudate (green), putamen (yellow) and nucleus accumbens (blue) according to the Harvard-Oxford subcortical structural atlas as implemented in conn.

982x358mm (39 x 39 DPI)

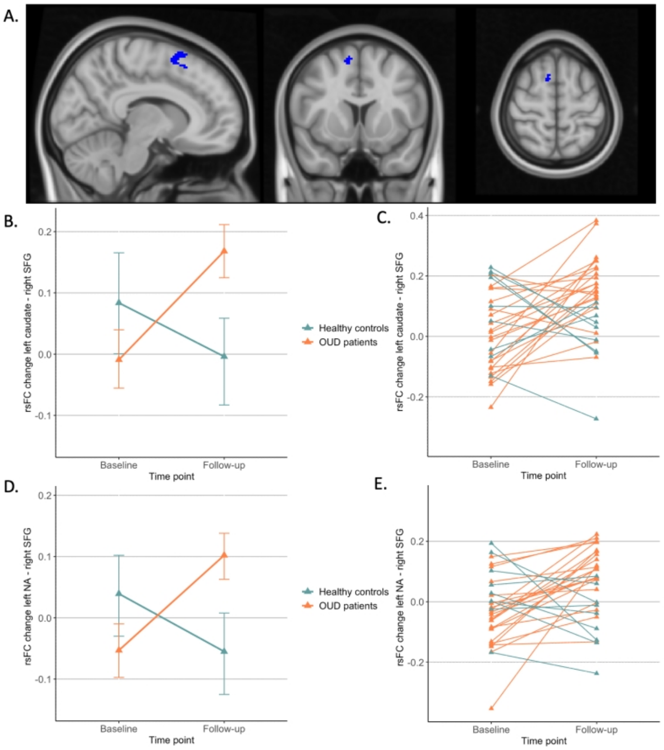

Figure 2. Resting-state functional connectivity (rsFC) from binge/intoxication regions to the right superior frontal gyrus (SFG). (A) Location of the cluster in right SFG (x=+10, y=+10, z=+60). Significant group*time interactions in rsFC from (B) left caudate with (C) individual trajectories and (D) left nucleus accumbens (NA) with (E) corresponding individual trajectories.

254x190mm (240 x 240 DPI)

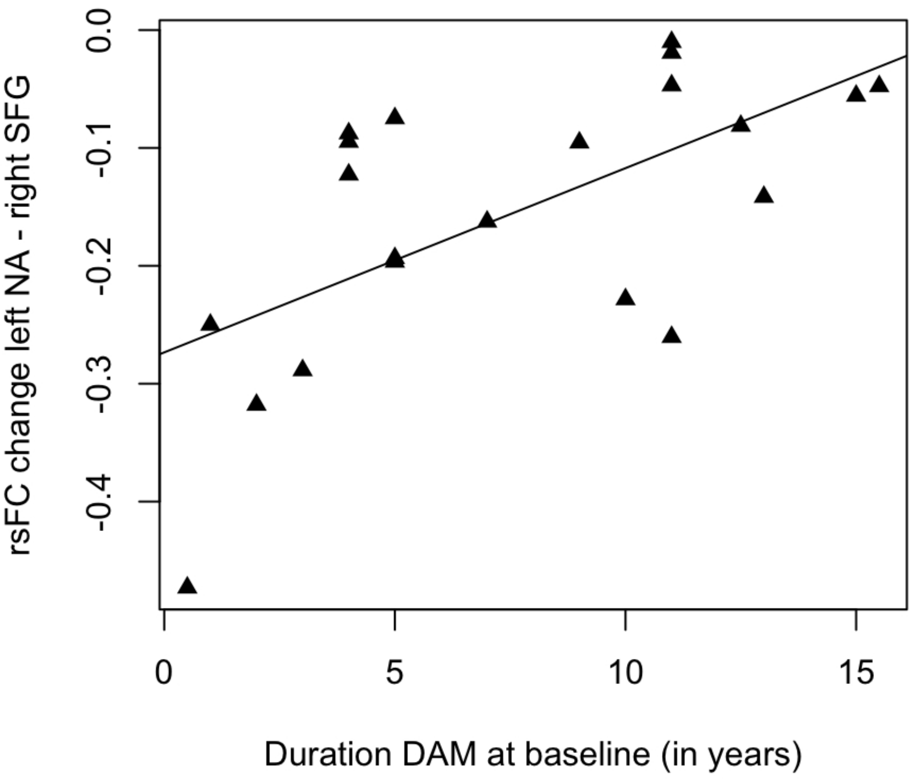

Fig3 - Significant positive correlation (r=0.63, p<.01) between resting-state functional connectivity (rsFC) changes in left nucleus accumbens (NA) to right superior frontal gyrus (SFG) and duration of DAM treatment (in years) in OUD patients.

332x320mm (72 x 72 DPI)

Table 1. Sociodemographic and behavioral characteristics of the study sample

	Patients (N=22)	Controls (N=9)	Comparison
Sociodemographic measures			
Age baseline, mean (SD)	42.32 (5.41)	39.33 (9.42)	T(10.23)=0.89, p=.39
Age follow-up, mean (SD)	50.95 (5.44)	48.22 (9.11)	T(10.42)=0.84, p=.42
Gender, male/female	16/6	5/4	χ2=0.26, p=.61
Handedness, right/left	22/0	9/0	-
Education, mean (SD)	10 (1.14)	13.44 (4.1)	T(8.62)=-2.48, p=.04
Smoking baseline, mean per day (SD)	22.23 (9.72)	12.22 (7.01)	T(20.65)=3.20, p<.01
Smoking follow-up, mean per day (SD)	17.32 (8.32)	5.33 (7.4)	T(16.72)=3.94, p<.01
Smoking, mean change (BL-FU) (SD)	4.91 (7.94)	6.89 (11.44)	T(11.3)=-0.47, p=.64
Benzodiazepine use baseline, yes/no	6/16	0/9	χ2=1.55, p=.21
Benzodiazepine use follow-up, yes/no	7/15	1/8	χ2=0.55, p=.46
Benzodiazepine use change (BL-FU)[a]	4/15/3	1/8/0	χ2=1.79, p=.41
Cocaine use baseline, yes/no	9/13	0/9	χ2=3.39, p=.07
Cocaine use follow-up, yes/no	10/12	0/9	χ2=4.14, p=.04
Cocaine use change (BL-FU)[a]	4/15/3	0/9/0	χ2=3.7, p=.16
Cannabis use baseline, yes/no	8/14	2/7	χ2=0.12, p=.73
Cannabis use follow-up, yes/no	8/14	1/8	χ2=0.94, p=.33
Cannabis use change (BL-FU)[a]	4/14/4	1/6/2	χ2=0.26, p=.88
Heroin related measures			
Age of first heroin use (years), mean (SD)	19.27 (3.4)	N.A.	N.A.
Duration heroin use baseline (years), mean (SD)	22.05 (5.55)	N.A.	N.A.
Duration DAM baseline (years), mean (SD)[b]	7.59 (4.64)	N.A.	N.A.
Daily heroin dose at BL, iv (mg), mean (SD)	345.91 (129.23)	N.A.	N.A.
Daily heroin dose at FU, iv (mg), mean (SD)	234.55 (182.44)	N.A.	N.A.
Daily heroin dose at FU, total (mg), mean (SD)[c]	351.59 (167.51)	N.A.	N.A.
Heroin dose change (BL-FU), iv (mg), mean (SD)	111.36 (211.73)	N.A.	N.A.
Clinical measures			
BDI baseline sum, mean (SD)	16.55 (7.89)	2.67 (4)	W=187.5, p<.001
BDI follow-up sum, mean (SD)	12.72 (9.04)	3.13 (5.03)	W=152.5, p<.01
BDI change (BL-FU), mean (SD)	3.82 (7.06)	-0.5 (3.07)	W=122, p=.12
HCQ baseline sum, mean (SD)	157 (25.37)	N.A.	N.A.
HCQ follow-up sum, mean (SD)	145.62 (57.89)	N.A.	N.A.
HCQ change (BL-FU), mean (SD)	11.95 (54.52)	N.A.	N.A.

Notes. DAM = Diacetylmorphine, BDI = Beck Depression Inventory, HCQ = Heroin craving questionnaire, N.A. = not applicable, BL = Baseline, FU = Follow-up.
[a] Baseline no and follow-up yes / no change / baseline yes and fallow-up no.
[b] 1 missing value.
[c] sum of intravenous dose and 0.5*per oral dose.

For Review Only
