# Supplementary material for: Striatal resting-state connectivity after long-term diacetylmorphine treatment in opioid-dependent patients
Source: Brain Commun. 2022 Oct 26;4(6):fcac275. doi: 10.1093/braincomms/fcac275 (PMC9642101; doi:10.1093/braincomms/fcac275)
Supplement: fcac275_Supplementary_Data [file fcac275_supplementary_data.zip › Supplementary material.docx]

**Striatal resting-state connectivity after long-term diacetylmorphine treatment in opioid-dependent patients**

Supplementary material

**Supplementary results**

**Supplementary Table 1. Detected clusters in the first half of the patient group compared to healthy controls.**

| **Brain region** | **MNI (x,y,z)** | **k** | **size p-FWE** | **size p-FDR** | **size p-unc** | **peak p-FWE** | **peak p-unc** |
| --- | --- | --- | --- | --- | --- | --- | --- |
| Superior frontal gyrus right | +10 +10 +60 | 21 | 0.691639 | 0.438651 | 0.015185 | 0.999920 | 0.000067 |
| Frontoorbital cortex right | +28 +22 -14 | 20 | 0.739512 | 0.438651 | 0.017362 | 0.998717 | 0.000043 |
| Planum temporale right | +66 -12 +06 | 15 | 0.934717 | 0.438651 | 0.035223 | 0.999966 | 0.000074 |
| Frontal pole left | -14 +52 -14 | 15 | 0.934717 | 0.438651 | 0.035223 | 0.785373 | 0.000008 |
| Superior frontal gyrus left | -10 +10 +68 | 14 | 0.958080 | 0.438651 | 0.040941 | 0.999759 | 0.000057 |
| Anterior superior temporal gyrus left | -62 -08 +04 | 14 | 0.958080 | 0.438651 | 0.040941 | 0.999220 | 0.000047 |
| Not labeled | -16 -18 -44 | 14 | 0.958080 | 0.438651 | 0.040941 | 0.994660 | 0.000032 |

Notes. K = cluster size in number of voxels, FWE = familywise error corrected, FDR = false discovery rate.

**Supplementary Table 2. Detected clusters in the second half of the patient group compared to healthy controls.**

| **Brain region** | **MNI (x,y,z)** | **k** | **size p-FWE** | **size p-FDR** | **size p-unc** | **peak p-FWE** | **peak p-unc** |
| --- | --- | --- | --- | --- | --- | --- | --- |
| Superior frontal gyrus right | +12 +10 +56 | 34 | 0.188553 | 0.141574 | 0.002574 | 0.717610 | 0.000006 |
| Not labeled | +18 -20 -40 | 19 | 0.758559 | 0.481475 | 0.017508 | 0.577042 | 0.000004 |
| Precentral gyrus left | -44 +02 +50 | 16 | 0.889408 | 0.497333 | 0.027127 | 0.965895 | 0.000018 |

Notes. K = cluster size in number of voxels, FWE = familywise error corrected, FDR = false discovery rate.
