# Supplementary material for: Striatal resting-state connectivity after long-term diacetylmorphine treatment in opioid-dependent patients
Source: Brain Commun. 2022 Oct 26;4(6):fcac275. doi: 10.1093/braincomms/fcac275 (PMC9642101; doi:10.1093/braincomms/fcac275)
Supplement: fcac275_Supplementary_Data [file Original_submission_decision_letter.pdf › Original_submission_decision_letter.pdf]

From: brcoms.editorialoffice@oup.com

To: annachiara.schaub@unibas.ch

CC:

BCC:

Subject: Brain Communications - Decision on Manuscript ID BRAINCOM-2022-089

Body: 09-Jun-2022

Dear Ms. Schaub,

Thank you for your recent submission BRAINCOM-2022-089 entitled "Striatal resting-state connectivity after long-term diacetylmorphine treatment in opioid-dependent patients" to Brain Communications.

Your manuscript has been reviewed, and while the reviewers and editorial team appreciate many aspects of the paper, they raise some concerns that you will need to address in order for the paper to be considered for publication.

If you can address these concerns, I invite you to submit a revised manuscript and a response to each of the reviewers' comments. The reviewer(s)' comments are included at the bottom of this message.

In addition to the reviewers' comments, please also address our Scientific Editor's comments added below to comply with our requirements for presentation of scientific data at Brain Communications.

Please format your revised manuscript according to our Instructions to Authors https://academic.oup.com/braincomms/pages/General_Instructions – a summary of key points is attached to help with this.

To revise your manuscript, click the link below or log into https://mc.manuscriptcentral.com/braincom and enter your Author Centre, where you will find your manuscript title listed under "Manuscripts with Decisions." Under "Actions," click on "Create a Revision." Your manuscript number will be appended to denote a revision.

*** PLEASE NOTE: This is a two-step process. After clicking on the link, you will be directed to a webpage to confirm. ***

https://mc.manuscriptcentral.com/braincom?URL_MASK=ea121d018074412e97f13e50d6da01c3

Please upload a revised manuscript with changes tracked with track changes or in coloured text.

When submitting your revised manuscript, you will be asked to respond to the reviewer(s) comments. Please be as specific as possible in your response to all of

the comments.

The editorial team at Brain Communications aims to increase transparency in scientific publishing and to demonstrate the value that peer review brings to the scientific process. Please therefore consider whether you are willing to have reviewer(s)' comments on your paper and your response published as supplementary data with the paper if it is accepted. This is entirely optional and will only be done if both the authors and reviewers agree. While submitting your revision there will be an opportunity to review or update the answer you gave previously, if necessary.

IMPORTANT: Your original files are available to you when you upload your revised manuscript. Please delete any redundant files before completing the submission.

Because we are trying to facilitate timely publication of manuscripts submitted to Brain Communications, we would appreciate your revised manuscript as soon as possible. If it is not possible for you to resubmit within 3 months, the option to submit a revision will expire, and we would then consider your revised paper as a new submission. However, if you need a little more time, please get in touch and we can extend this deadline for you.

Once again, thank you for submitting your manuscript to Brain Communications and I look forward to receiving your revision.

Best wishes

Prof. Nicola Pavese
Associate Editor, Brain Communications

Professor Tara Spires-Jones
Founding Editor, Brain Communications

Reviewer(s)' Comments to Author:

Reviewer: 1 (Xiu-Xia Xing)

Comments to the Author
There are several major issues coming up during my reading of the manuscript.

1. Reproducibility: The sample sizes are really limited. Is there any way of getting such novel and important findings to be validated regarding their reproducibility. This seems highly relevant and critical in terms of the recent concerns raised by the field (see https://www.nature.com/articles/d41586-022-00767-3). The authors could try to figure out a way of doing an validation by either using a new sample or

split their samples into different parts although this can be challenging according this clinical research. The sample size is relatively small and thus important to claim the limitation of reproducibility on the present findings. Meanwhile, the treatment on/off or other within-subject design may not need such big sample size. This should be discussed in terms of the experimental design and statistical methods employed by the present work.

2. Measurements: A fundamental factor of measuring interindividual differences is reliability (see why in https://www.nature.com/articles/s41562-019-0655-x). This one is highly related to the sample size and effect size issue, and thus must be considered by introducing the test-retest reliability of the measurements employed in the present work (e.g., the seed-based FC measures), and discussing about reliability challenges or limitations at the current stage (see https://pubmed.ncbi.nlm.nih.gov/31494250/). Please include the reliability of the related measurements.

3. Statistics: All the reports of statistics must be acommpanied with statistical power, effect size, confidence interval and corrections if multiple comparisons done.

4. Study-Specific Issues: The seminal work on the striatum-seeded FC has been done in normal people but seems no credit in the present work (see https://pubmed.ncbi.nlm.nih.gov/18400794/). It would be more comprehensive if the authors can demonstrate the whole-brain RSFC maps for both patient and control groups. as well as before and after the treatment. The correlation between the duration DAM and rsFC was very high (than the test-retest reliability of rsFC, kind of puzzling outcomes regarding the up-bound of rsFC as its reliability, again see https://www.nature.com/articles/s41562-019-0655-x).

Reviewer: 2

Comments to the Author
The authors report changes in striatal resting-state connectivity after nine-year treatment with diacetylmorphine in 22 opioid-dependent patients. Nine healthy controls were also investigates. All the participants in the follow-up study had baseline MRI studies and the results have previously been reported.

I think the results are interesting but unfortunately, as the authors also discuss in the limitation section of the discussion, the study groups are not balanced. I am not sure why they decided to use the same controls as in the baseline study. A new, more numerous, healthy control group age-matched with the follow-up group or even a hybrid group with the original controls and new recruited individual would have strengthened the results. I would encourage the authors to try to include more healthy controls.

Comments from the Scientific Editor:

- Fig2b-e: please report in the figure legend the test used and relevant test statistic values alongside p values (for example F or t values for ANOVAs and t-tests).

-Please supply a graphical abstract. A graphical abstract is a visual representation of the central finding or methodology of the paper. It will be displayed in the table of contents. Authors should strive to make the graphical abstract informative, interesting, visually appealing, and straightforward (no caption needed, no abbreviations except gene names). Please upload as a separate high resolution image file (tiff preferred). The technical requirements for the graphical abstracts are:

--size of the submitted image: 1200 pixels square at 300dpi

--font of the text: Arial 12-16 point (smaller fonts will not be legible online)

-Please add an Abbreviated Summary (up to 50 words) of your paper that captures the main purpose and conclusions of the work. The summary should state the main results written in the third person (‘[1st author surname] et al. report that …’) and conclusion. This summary will be used in the contents list online and should not contain any abbreviations other than those on the accepted abbreviations list and accepted gene/protein names, making it accessible to the non-specialist. Please upload it as a Word document. Allowed abbreviations: AIDS; ANOVA; AMPA; ATP; A,T,C,G; CNS; CSF; CT; DNA; ECG; EEG; EMG; GABA; HIV; MRI; NMDA; PET; PCR; RNA.

-Please include a paragraph about statistical analysis in the 'Materials and Methods' section.
