# Supplementary material for: Striatal resting-state connectivity after long-term diacetylmorphine treatment in opioid-dependent patients
Source: Brain Commun. 2022 Oct 26;4(6):fcac275. doi: 10.1093/braincomms/fcac275 (PMC9642101; doi:10.1093/braincomms/fcac275)
Supplement: fcac275_Supplementary_Data [file Original_submission_Response_to_reviewers_comments.pdf › Original_submission_Response_to_reviewers_comments.pdf]

We would like to thank all reviewers for their comments, which have helped us to improve the paper. We provide a point-by-point reply below.

Reviewer(s)' Comments to Author:

Reviewer: 1

Comments to the Author
There are several major issues coming up during my reading of the manuscript.

1. Reproducibility: The sample sizes are really limited. Is there any way of getting such novel and important findings to be validated regarding their reproducibility. This seems highly relevant and critical in terms of the recent concerns raised by the field (see https://www.nature.com/articles/d41586-022-00767-3). The authors could try to figure out a way of doing an validation by either using a new sample or split their samples into different parts although this can be challenging according this clinical research. The sample size is relatively small and thus important to claim the limitation of reproducibility on the present findings. Meanwhile, the treatment on/off or other within-subject design may not need such big sample size. This should be discussed in terms of the experimental design and statistical methods employed by the present work.

Response:

Thank you very much for pointing out that important concern. We conducted an additional analysis with the approach you suggested and split the patient group into two different samples. Analyses were then conducted separately for both groups compared to the healthy control sample.

The methods are described on page 8:

"To further validate our findings, we conducted an additional analysis by splitting the sample of patients into two separate samples (half 1: 1 0 1 0 1 0 etc., half 2: 0 1 0 1 0 1 etc.) and run two separate analyses comparing both subgroups with the healthy controls including the same covariates (2x2 mixed ANCOVA)."

With the standard settings for cluster-based inferences (cluster-size p-FDR<.05, voxel p-uncorrected<.001) no significant clusters were detected. However, when setting the cluster threshold to p-uncorrected<.05, multiple significant clusters were detected. In both subanalyses, the most prominent cluster was again located in the superior frontal gyrus.

The results of these additional analyses were now reported on page 9 in the revised manuscript:

"In the additional analyses with the two subsamples of patients, the cluster in the SFG was detected in both subsamples after adapting the cluster size threshold to p-uncorrected<.05 (see Supplementary Tables 1 and 2 for details on the detected clusters)."

Details of these analyses were included in the supplement.

Although the cluster in the superior frontal gyrus were only prevalent in the adapted settings for

cluster-based inferences, we see this as a confirmation of our results.

We also adjusted the limitation section on page 13 to address your important concern about reproducibility:

“We were able to include data from a healthy control sample to exclude general aging effects. However, the study groups were not balanced and relatively small; the control group consisted of only nine subjects. Several participants of the original study were lost to follow-up. Nevertheless, it is a strength of the study that we were able to include 22 of the original 29 DAM patients, a population very difficult to follow-up for longer periods. With our rather small sample, the reproducibility of our results may be questioned although we were able to reproduce our results with adapted threshold in split samples. Generally, the reliability and reproducibility of actual neuroimaging analyses using MRI data is challenging. As subcortical seeds are associated with low reliability, our interest in seeds related to the binge state of addiction and the long test-retest interval may be sources of reduced reliability in comparison to shorter test-retest intervals and cortical seeds. Furthermore, the data acquisition of 5 min was relatively short. With short scan durations discomfort in the scanner and movement can be decreased, but it reduces the reliability of the scan and limits corrections in the preprocessing such as the exclusion of first scans. The results should be interpreted with these caveats in mind.”

2. Measurements: A fundamental factor of measuring interindividual differences is reliability (see why in https://www.nature.com/articles/s41562-019-0655-x). This one is highly related to the sample size and effect size issue, and thus must be considered by introducing the test-retest reliability of the measurements employed in the present work (e.g., the seed-based FC measures), and discussing about reliability challenges or limitations at the current stage (see https://pubmed.ncbi.nlm.nih.gov/31494250/). Please include the reliability of the related measurements.

Response:

We expanded the limitations related to the reliability in our study.

“We were able to include data from a healthy control sample to exclude general aging effects. However, the study groups were not balanced and relatively small; the control group consisted of only nine subjects. Several participants of the original study were lost to follow-up. Nevertheless, it is a strength of the study that we were able to include 22 of the original 29 DAM patients, a population very difficult to follow-up for longer periods. With our rather small sample, the reproducibility of our results may be questioned although we were able to reproduce our results with adapted threshold in split samples. Generally, the reliability and reproducibility of actual neuroimaging analyses using MRI data is challenging. As subcortical seeds are associated with low reliability, our interest in seeds related to the binge state of addiction and the long test-retest interval may be sources of reduced reliability in comparison to shorter test-retest intervals and cortical seeds. Furthermore, the data acquisition of 5 min was relatively short. With short scan durations discomfort in the scanner and movement can be decreased, but it reduces the reliability of the scan and limits corrections in the preprocessing such as the exclusion of first scans. The results should be interpreted with these caveats in mind.”, page 13.

3. Statistics: All the reports of statistics must be acommpanied with statistical power, effect size,

confidence interval and corrections if multiple comparisons done.

Response:

Thank you for the valuable input, we adapted multiple parts regarding these open points in our methods and results.

For the resting-state functional connectivity analysis, the joint inclusion of all seeds-of-interest was applied to avoid problems of multiple testing. We specified the approach in the methods on page 8:

"All six seeds of the binge stage were jointly included as sources to avoid problems of multiple testing;"

Due to the exploratory nature of the correlation analyses, corrections of multiple comparisons were not applied. We added a specification on page 8:

"As this analysis is of exploratory nature, no corrections for multiple testing were applied."

Regarding effect sizes and confidence intervals, we added them in the results and described the approach in the methods.

"Effect sizes were based on cohens' D or Cliffs delta for ordinal data.", page 8.

In the results on page 9, reports of statistics were adapted.

"The mean intravenous dose dropped significantly at the follow-up ($t(21)=2.47$, $p<0.05$, $d=0.7$, 95%-confidence interval (CI) -0.2-1.57), but when combining intravenous and oral doses at follow-up, the dose was not significantly different to the initial dose at baseline ($t(21)=-0.11$, $p=0.91$, $d=0.04$, 95-CI: -0.8- 0.87). Depressive symptoms in the patients decreased over the study period ($V=163.5$, $p<0.5$, delta=0.28, 96%-CI: -0.09-0.58), while craving (HCQ) did not change significantly ($V=139.5$, $p=0.41$, delta=0.06, 96%-CI: -0.29-0.4). Depressive symptoms in healthy controls did not change over time ($V=9$, $p=.44$, delta=-0.04, 96%-CI: -0.51-0.45)."

4. Study-Specific Issues: The seminal work on the striatum-seeded FC has been done in normal people but seems no credit in the present work (see https://pubmed.ncbi.nlm.nih.gov/18400794/). It would be more comprehensive if the authors can demonstrate the whole-brain RSFC maps for both patient and control groups. as well as before and after the treatment. The correlation between the duration DAM and rsFC was very high (than the test-retest reliability of rsFC, kind of puzzling outcomes regarding the up-bound of rsFC as its reliability, again see https://www.nature.com/articles/s41562-019-0655-x).

Response:

Thank you very much for the suggestion of this interesting paper from Di Martino et al., 2008. We included the association between the dorsal caudate and the superior frontal gyrus in our discussion as follows:

"Functionally related activity between the dorsal caudate and frontal regions including the superior

frontal gyrus have been detected in a seminal work in healthy participants.", page 11.

We furthermore expanded the limitations pointing to the relevance of these fronto-striatal connectivity in cognition in healthy controls and patients with addiction:

"We could not find any relations of fronto-striatal connectivity changes with behavioral measures such as craving, and data was not available for cognition related behaviors. As the detected regions are highly linked to cognitive control and response inhibition in healthy controls and patients with addiction, future studies may include further behavioral and cognitive measures to disentangle the clinical relevance of the findings.", page 13.

Related to the second aspect of the comment, we conducted two analyses on whole-brain network measures at the voxel level: an analysis on global correlation (average of correlation coefficients between each individual voxel and all of the voxels in the brain) and local correlation (average of correlation coefficients between each individual voxel and a region of neighboring voxels).
When using the same model (2x2 mixed ANCOVA) we did not find any interactions (cluster size p-FDR $p<.05$, voxel p-uncorrected $p<.001$).
These results point out that changes are specifically located in the striatum and not in the whole brain.

In the limitations we expanded the part on the reliability as follows:

"We were able to include data from a healthy control sample to exclude general aging effects. However, the study groups were not balanced and relatively small; the control group consisted of only nine subjects. Several participants of the original study were lost to follow-up. Nevertheless, it is a strength of the study that we were able to include 22 of the original 29 DAM patients, a population very difficult to follow-up for longer periods. With our rather small sample, the reproducibility of our results may be questioned although we were able to reproduce our results with adapted threshold in split samples. Generally, the reliability and reproducibility of actual neuroimaging analyses using MRI data is challenging. As subcortical seeds are associated with low reliability, our interest in seeds related to the binge state of addiction and the long test-retest interval may be sources of reduced reliability in comparison to shorter test-retest intervals and cortical seeds. Furthermore, the data acquisition of 5 min was relatively short. With short scan durations discomfort in the scanner and movement can be decreased, but it reduces the reliability of the scan and limits corrections in the preprocessing such as the exclusion of first scans. The results should be interpreted with these caveats in mind.", page 13.

Reviewer: 2

Comments to the Author
The authors report changes in striatal resting-state connectivity after nine-year treatment with diacetylmorphine in 22 opioid-dependent patients. Nine healthy controls were also investigates. All the participants in the follow-up study had baseline MRI studies and the results have previously been reported.

I think the results are interesting but unfortunately, as the authors also discuss in the limitation section of the discussion, the study groups are not balanced. I am not sure why they decided to use

the same controls as in the baseline study. A new, more numerous, healthy control group age-matched with the follow-up group or even a hybrid group with the original controls and new recruited individual would have strengthened the results. I would encourage the authors to try to include more healthy controls.

Response:

Thank you very much for your feedback on our study.
Due to the prospective nature of the study, it was only possible to use data from patients and healthy controls that participated in the baseline study. Unfortunately, the follow-up rate of healthy controls was not as high as in the patients. We like to note that recruitment in a 9-year follow-up study is demanding. While patients were still involved in the DAM maintenance program and as such more easily to recruit, many healthy control subjects were not reachable anymore with the previously received contact information.

With additional analyses, however, we were able to replicate our findings in more balanced groups as well. These additional analyses included subsamples of the patient group to test the validity of our results, see revised methods on page 8 and results on page 9. We were able to confirm the cluster in the superior frontal gyrus in both separate analyses which both included each 11 patients vs 9 controls.

Nevertheless, we completely agree that the sample size is a crucial limitation. We therefore adapted our limitations and expanded the point on the sample size on page 13.

"We were able to include data from a healthy control sample to exclude general aging effects. However, the study groups were not balanced and relatively small; the control group consisted of only nine subjects. Several participants of the original study were lost to follow-up. Nevertheless, it is a strength of the study that we were able to include 22 of the original 29 DAM patients, a population very difficult to follow-up for longer periods. With our rather small sample, the reproducibility of our results may be questioned although we were able to reproduce our results with adapted threshold in split samples. Generally, the reliability and reproducibility of actual neuroimaging analyses using MRI data is challenging."

Comments from the Scientific Editor:

- Fig2b-e: please report in the figure legend the test used and relevant test statistic values alongside p values (for example F or t values for ANOVAs and t-tests).

Response: Statistical values were added to the figure.

-Please supply a graphical abstract. A graphical abstract is a visual representation of the central finding or methodology of the paper. It will be displayed in the table of contents. Authors should strive to make the graphical abstract informative, interesting, visually appealing, and straightforward (no caption needed, no abbreviations except gene names). Please upload as a separate high resolution image file (tiff preferred). The technical requirements for the graphical abstracts are:
--size of the submitted image: 1200 pixels square at 300dpi
--font of the text: Arial 12-16 point (smaller fonts will not be legible online)

Response: We created a graphical abstract and submit it with the revision.

-Please add an Abbreviated Summary (up to 50 words) of your paper that captures the main purpose and conclusions of the work. The summary should state the main results written in the third person ('[1st author surname] et al. report that …') and conclusion. This summary will be used in the contents list online and should not contain any abbreviations other than those on the accepted abbreviations list and accepted gene/protein names, making it accessible to the non-specialist. Please upload it as a Word document. Allowed abbreviations: AIDS; ANOVA; AMPA; ATP; A,T,C,G; CNS; CSF; CT; DNA; ECG; EEG; EMG; GABA; HIV; MRI; NMDA; PET; PCR; RNA.

Response: We added an Abbreviated Summary and submit it with the revision.

-Please include a paragraph about statistical analysis in the 'Materials and Methods' section.

Response: The titles in the 'Materials and Methods' section were adapted and include now a title 'Statistical analysis'.
